# Molecular characterisation of four double-flowered mutants of *Silene dioica* representing four centuries of variation

**DOI:** 10.1093/jxb/erv139

**Published:** 2015-04-15

**Authors:** Elizabeth K. S. Ingle, Philip M. Gilmartin

**Affiliations:** ^1^School of Biological Sciences, University of East Anglia, Norwich Research Park, Norwich NR4 7TJ, UK; ^2^John Innes Centre, Norwich Research Park, Colney Lane, Norwich NR4 7UH, UK

**Keywords:** C-function MADS-box, dioecy, double-flowered, floral homeotic mutation, sex determination, *Silene dioica*.

## Abstract

Records of double-flowered *Silene dioica* date from the late sixteenth century and four named varieties are grown today, as previously, for their horticultural interest. Although double-flowered mutants have been characterized in several plants, their study in dioecious species is of particular interest due to influences of the homeotic mutation on the different floral whorl configurations in males and females. We have analysed four double-flowered varieties of *Silene dioica*: Flore Pleno and Rosea Plena date back to the seventeenth and nineteenth centuries, Thelma Kay and Firefly were recognized in the latter part of the twentieth and early twenty-first centuries. We have analysed the floral structure of the four varieties, which have distinct floral architectures. Based on Y chromosome-specific PCR analysis we show that Firefly is male and that the other three varieties are female: Random Amplification of Polymorphic DNA (RAPD) analyses suggested a common origin for the three female varieties. The double-flowered phenotype in all four varieties is caused by mutation of the C-function MADS-box transcription factor gene *SDM1*. We show that Firefly carries a unique 44bp insertion into *SDM1,* revealing an independent origin for this variety. Comparative analysis of *SDM1* cDNA and genomic sequences in Flore Pleno, Rosea Plena and Thelma Kay shows that all three are caused by the same 7bp insertion within *SDM1* and therefore share a common origin. The three alleles also differ by several single nucleotide polymorphisms, which represent somatic mutations accumulated over four centuries of asexual propagation.

## Highlight

Four double-flowered mutants of dioecious *Silene dioica* have been characterized; we recount their 400 year history, trace their ancestral genetic relationships, describe their phenotypes, and characterize mutant MADS-box gene alleles.

## Introduction

*Silene dioica* is a dioecious species with male and female flowers produced on separate plants. Sex is determined by a dominant Y chromosome, which suppresses carpel development in XY male flowers and is required for development and maturation of the stamens. Male flowers have five sepals, five petals and 10 stamens with an arrested fourth floral whorl. In female XX flowers the stamen primordia form but arrest early in development and in the absence of the Y chromosome, five fused carpels develop to maturity. Female flowers therefore have five sepals, five petals, 10 arrested stamen primordia and five fused carpels (Westergaard, 1958; Grant *et al.*, 1994; Farbos *et al.*, 1997; Scutt *et al.*, 1997*b*). Numerous studies have focused on the identification of male-specific genes in *S. dioica* and its close relative *S. latifolia*, with the objective of understanding the molecular basis of sex determination (Grant *et al.*, 1995; Barbacar *et al.*, 1997; Matsunaga *et al.*, 1997; Robertson *et al.*, 1997; Scutt and Gilmartin, 1998; Scutt *et al.*, 2002; Blavet *et al.*, 2011). Extensive work has been done to try and identify Y chromosome-encoded genes (Scutt *et al.*, 1997*a*; Delichere *et al.*, 1999; Matsunaga *et al.*, 1999; Sugiyama *et al.*, 2002; Hobza *et al.*, 2004; Cegan *et al.*, 2010) and also to explore sex chromosome evolution (Guttman and Charlesworth, 1998; Filatov and Charlesworth, 2002; Lengerova *et al.*, 2004; Kaiser *et al.*, 2009; Nishiyama *et al.*, 2010). However, the key sex determination genes remain to be identified and characterized.

Double flowers have been cultivated for their aesthetic value since the earliest days of horticulture and their analysis in model systems, such as *Arabidopsis thaliana* and *Antirrhinum majus*, led to the development of the ABC model of flower development (Coen and Meyerowitz, 1991), which has since been expanded to include other gene functions and reflect gene duplications (Davies *et al.*, 1999; Theissen, 2001; Airoldi and Davies, 2012; Heijmans *et al.*, 2012; Mach, 2012). C-function MADS-box genes have been functionally characterized in several species and are required for correct development of the third and fourth floral whorls, which form stamens and carpels respectively. Examples include *Arabidopsis* (Yanofsky *et al.*, 1990), *Antirrhinum* (Bradley *et al.*, 1993), *Petunia* (Kater *et al.*, 1998) and tomato (Pnueli *et al.*, 1994) as well as monoecious cucumber (Kater *et al.*, 1998). Mutation of C-function genes leads to development of double flowers with multiple petals and no reproductive structures. Analysis of double-flowered mutants in *S. dioica* provides the opportunity to examine interactions between the genes controlling sex determination and those controlling floral organ identity and floral whorl number.

**Fig. 1.**
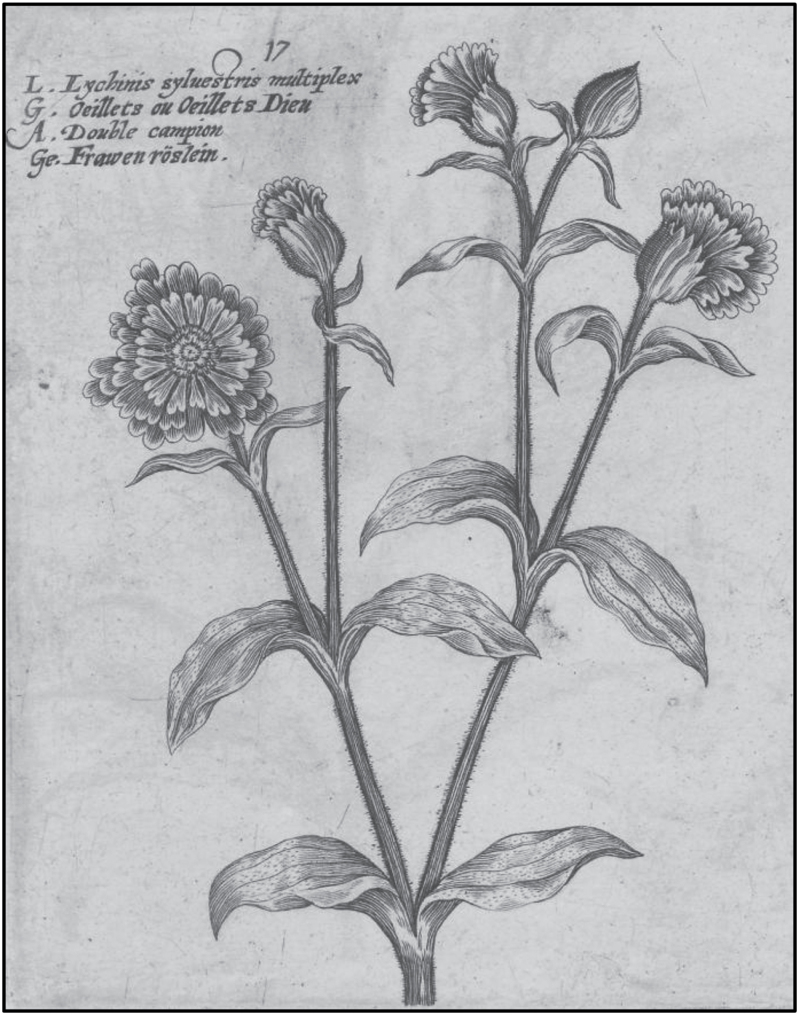
Seventeenth century engraving of double-flowered *S. dioica.* Engraving of double-flowered *S. dioica* from *Hortus Floridus, Altera Pars* (van de Passe, 1605) courtesy of Utrecht University Library.

In Book IV of his *Enquiry into Plants* from the fourth century bc, Theophrastus describes the multi-petalled double-flowered roses, which differ from the normal five-petalled varieties, remarking on the existence of some that are ‘hundred-petalled’ (Hort, 1980). More recent records of double flowers from the seventeenth century include double-flowered *S. dioica* and *S. latifolia* plants (van de Passe, 1605; Besler, 1613; Parkinson, 1629). Fig. 1 shows a copper-plate engraving of double red campion (*S. dioica*) from *Hortus Floridus Altera Pars* (van de Passe, 1605). Although the exact publication date is uncertain (Gerard, 1996), this engraving possibly represents the earliest image of double-flowered *S. dioica*. Double rose campion, *Lychnis coronaria* was depicted in the first edition of Gerard’s *Herbal* (Gerard, 1597) although double-flowered *L. sylvestris* (now *S. dioica*) were not, there is however documentary evidence that Gerard grew double-flowered *S. dioica* in his garden (Jackson, 1876). Double white campion *S. latifolia* is also described in early texts (Besler, 1613) but it is no longer cultivated, although it was available until the 1830s (Loudon, 1830). Early records refer to double-flowered *S. dioica* as *L. sylvestris* multiplex or ‘Bachelor’s Buttons’. There are now four varieties of double-flowered *S. dioica* in cultivation: Flore Pleno, Rosea Plena, Thelma Kay and Firefly.

Records from later herbals and florilegia (Parkinson, 1629; Weinmann, 1737; Curtis, 1777), refer to double red campion as *S. dioica* Flore Pleno, a name first used by Besler (Besler, 1613) and this variety has been in cultivation ever since. The double-flowered mutation renders the plant sterile and asexual propagation provides a direct link between contemporary plants and the original mutant. The origin of the double-flowered variety is recorded as having been developed from single red campion grown in gardens in England: ‘The double varieties are exceedingly ornamental, the flowers large, very double … the single red sort grows wild … in many parts of England, from which the doubles were accidentally obtained by culture in gardens.’ (Encyclopedia Perthensis, 1816).

The name Flore Pleno has been in use since 1613 (Besler, 1613), but in 1837 another name describing double *S. dioica* appeared in the literature when MD Henrard, a member of the Société d’Horticulture de Liége, exhibited *S. dioica* Rosea Plena at the twelfth Society summer exhibition (Anon., 1837). He is described as secretary of the Society with an interest in new varieties and double flowers. As this is the earliest recorded use of this new name, it seems likely that Rosea Plena dates from the mid-1830s. Thelma Kay was described in 1995 in the journal of the Hardy Plant Society (Brown, 1996) as a new variegated form of double *S. dioica* that arose spontaneously in a population of double *S. dioica* in the Manchester garden of Thelma Kay, after whom it was named. It is not documented whether the progenitor was Flore Pleno or Rosea Plena. The origin of Firefly is the best documented of the four cultivars. Plant Breeders Rights were granted to this new variety in 2009; this plant arose in a population of wild-type *S. dioica* cultivated in Woodbridge, Suffolk.

Although there is a well-documented history of double-flowered mutants of *S. dioica*, none have been characterized at a molecular level, although a putative C-function MADS-box gene, *SLM1,* has been characterized in *S. latifolia* (Hardenack *et al.*, 1994). *SLM1* is the only predicted C-function gene in *S. latifolia*; previous studies investigated the localization but not the function of the gene and did not define a mutant phenotype associated with the locus. *SLM1* showed the typical expression profile of C-function MADS-box gene in the third and fourth floral whorls of early flower buds and no difference was seen in expression between the sexes (Hardenack *et al.*, 1994). However, differences in expression were observed between male and female flowers for the predicted B-function MADS-box genes, *SLM2* and *SLM3,* which are expressed in the fourth whorl of female but not male flowers. Previous studies of a double-flowered *S. latifolia* mutant, *Sl-dfl*, generated by gamma irradiation of pollen (Scutt *et al.*, 1999) showed reduced expression of the full length transcript for the putative MADS-box gene *SLM1* in pre-meiotic flower buds but did not explore whether the *SLM1* coding region contained any mutations.

Here we define the function of *SDM1*, the *S. dioica* homologue of *SLM1,* and unite this with the history of double-flowered *S. dioica* to investigate the basis of these homeotic mutants in this dioecious species. We have characterized and compared flower architecture in the four available double varieties of *S. dioica* and identified mutations in *SDM1* responsible for the double-flowered phenotypes, which we discuss in relation to dioecious flower development. Molecular differences between the four *S. dioica* double-flowered mutants have revealed insights into molecular relationships spanning four centuries.

## Materials and methods

### Source of plant material

Wild-type *S. dioica* was obtained from a wild population at the University of East Anglia. We sourced Flore Pleno from Ravensthorpe Nurseries, Northampton, UK, Rosea Plena from The Herb Nursery, Rutland, UK and Firefly from West Country Nurseries, Bideford, UK. Thelma Kay has been grown as part of our laboratory plant collection. Plants were grown as described previously (Scutt *et al.*, 1997*b*).

### Analysis of *SDM1* genomic DNA and cDNA sequences

Genomic DNA was isolated from leaf tissue using a Nucleon Phytopure Genomic DNA Extraction kit. RNA was isolated from unopened flower buds using an Ambion RNaqueous kit and cDNA was synthesized with MMLV reverse transcriptase according to the supplier’s instructions. Illumina sequencing was performed at The Genome Analysis Centre, Norwich, using *S. dioica* genomic DNA which yielded 18 169 313 291 paired-end reads which were then assembled into 18 321 540 sequences using ABySS v1.3.4 (Simpson *et al.*, 2009) (k-mer length=71) with an N50 of 146, average sequence length 142bp (minimum 71bp, maximum 84 125bp). Sex-specific PCR analysis used primers for male-specific *Men-153* (Jenkins, 2002; Scutt *et al.*, 2002) with genomic DNA template and Go-Taq Flexi. PCR amplification used an initial denaturation step at 94ºC for 2min, followed by 35 cycles at 94ºC for 55 s, 55ºC for 55 s and 72ºC for 55 s, and a final extension stage at 72ºC for 5min. Primers 5ʹ-ACACCCCCAAATCAGGTATGTTAT-3ʹ and 5ʹ-GCTACTGGTGTCACTTATTCCATTAA-3ʹ were used to amplify the ~550bp Y-chromosome specific product. The 200bp autosomal control band was amplified using primers 5ʹ-AGGGCTAGTC ACAAGAAAGTG-3ʹ and 5ʹ-TCCGTACTCTAATTGTAATGT-3ʹ.

*SDM1* was amplified from genomic DNA in overlapping sections using Phusion high-fidelity polymerase (New England Biolabs) with primers designed to *SDM1* sequences obtained from a *de novo* assembly of Illumina paired-end sequences from *S. dioica* genomic DNA. PCR amplification used an initial denaturation step at 98ºC for 2min, 30 cycles of 98ºC for 10 s, 50ºC for 20 s and 72ºC for 1min, and final extension stage at 72ºC for 10min.

*SDM1* and *SLM1* cDNA was amplified using Go-Taq Flexi with primers 5ʹ-ATGGAGTTTTCAAGCCAAATTAC-3’ and 5ʹ-TTAGTTAAGCTGGAGAGTTGTC-3ʹ based on the coding sequence of *SLM1* (Hardenack *et al.*, 1994) with an initial denaturation step at 95ºC for 2min 30 s, 35 cycles of 95ºC for 45 s, 52ºC for 45 s and 72ºC for 45 s, and a final extension stage at 72ºC for 5min. 50ng cDNA used per 50 µl reaction mix.

### Random Amplification of Polymorphic DNA

RAPD primers were obtained from MWG Operon and PCR was carried out using 50ng genomic DNA per 25 µl Go-Taq Flexi reaction mix with an initial denaturation step at 95ºC for 5min, 45 cycles of 95ºC for 1min, 31ºC for 1min and 72ºC for 1min, and a final extension stage at 72ºC for 10min.

### Analysis of flowers and petals

Whole flower photographs and petal number counts used flowers in full bloom at the point when the sepal had split open and, in the case of Thelma Kay, Rosea Plena and Flore Pleno, when the outermost petals had fallen back to lie at 90 degrees to the stem. Ten flowers of each phenotype were counted (Supplementary Table S1) and mean organ number and standard error was calculated for each phenotype (Table 1).

**Table 1.** Average floral organ number per flower in double-flowered varieties of *S. dioica* Values show mean counts from 10 flowers, standard errors are shown in parentheses. Not applicable, n/a.

**Variety**	**Outer petals**		**Extra sepals**	**Middle petals**		**Extra sepals**	**Inner petals**		**Extra sepals**	**Whole flower**		**Total petals**	**Total organs**
	Pink	White		Pink	White		Pink	White		Pink	White		
Wild-type	5.0 (0)	n/a	n/a	n/a	n/a	n/a	n/a	n/a	n/a	5.0 (0)	n/a	5.0 (0)	5.0 (0)
Flore Pleno	47.3 (1.9)	2.1 (1.0)	5.0 (0)	4.4 (2.0)	21.1 (5.7)	5.0 (0)	0.0	17.3 (1.6)	n/a	51.7 (3.4)	40.5 (5.6)	92.2 (5.2)	102.2 (5.2)
Rosea Plena	50.3 (1.4)	2.5 (1.1)	5.0 (0)	6.5 (2.2)	14.2 (1.2)	5.0 (0)	0.0	18.0 (2.3)	n/a	56.8 (3.3)	34.7 (3.8)	91.5 (4.1)	101.5 (4.1)
Thelma Kay	61.4 (4.8)	40.3 (2.6)	n/a	n/a	n/a	n/a	n/a	n/a	n/a	61.4 (4.8)	40.3 (2.6)	101.7 (3.6)	101.7 (3.6)
Firefly	32.6 (1.2)	0.7 (0.3)	n/a	n/a	n/a	n/a	n/a	n/a	1.0 (0)	32.6 (1.2)	0.7 (0.3)	33.3 (1.5)	34.3 (1.5)

## Results

### Comparative phenotypic analysis of four double-flowered mutants

**Fig. 2.**
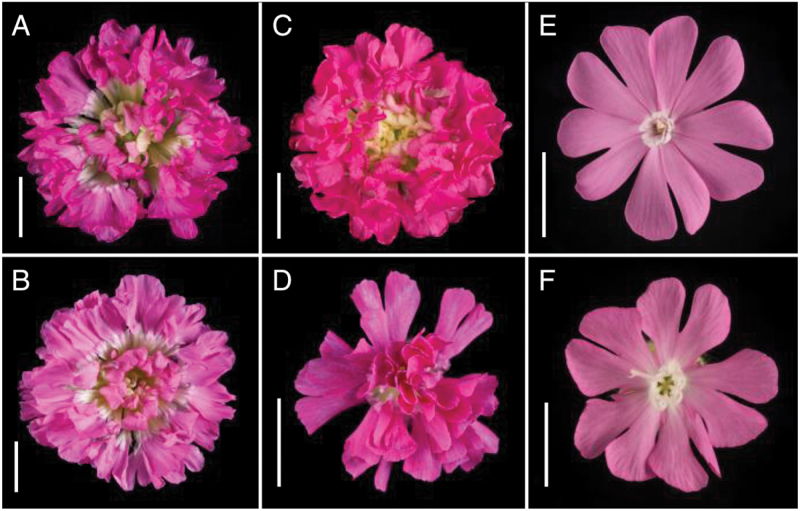
Photographic images of flowers from double-flowered varieties of *S. dioica*. (A) Flore Pleno, (B) Rosea Plena, (C) Thelma Kay, (D) Firefly, in comparison to (E) wild-type male and (F) wild-type female (F). Scale bars =1cm.

Examination of the floral phenotype of the four double-flowered varieties of *S. dioica*—Flore Pleno, Rosea Plena, Thelma Kay and Firefly—shows that all varieties have increased petal number but their flower structures are not identical. Fig. 2 shows individual flowers from the four varieties alongside wild-type male and female flowers. In all four double-flowered varieties, in addition to increased petal number, stamens and carpels are absent. The homeotic conversion of these whorls, and increased organ number, results in a generally disordered flower structure with no distinct whorls present after the sepals. The flowers also include some white petals, which are not found in wild-type. The engraving from *Hortus Floridus Altera Pars* (van de Passe, 1605) (Fig. 1) provides an early insight into the mutant phenotype and the similarities to the flowers of modern day variety Flore Pleno (Fig. 2A) are clearly visible, including the organization of petals into sections or rings.

**Fig. 3.**
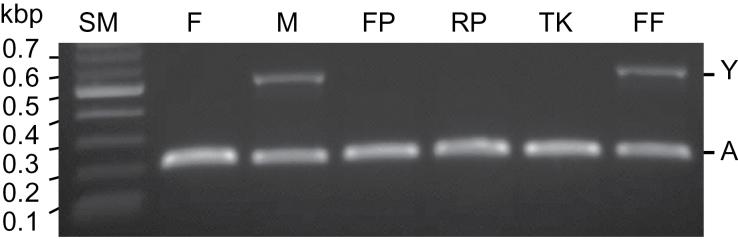
PCR analysis of genomic DNA from Flore Pleno (FP), Rosea Plena (RP), Thelma Kay (TK) and Firefly (FF) using primers for an Y chromosome-specific marker (Y) and autosomal controls locus (A) (Jenkins 2002). Female (F) and male (M) wild-type controls also shown. Size markers (SM) are shown in kb.

In addition to differences in floral morphology there are differences in flower size. In Flore Pleno, Rosea Plena and Thelma Kay the flowers are larger than those of wild-type and Firefly (Fig. 2). In dioecious *Silene* species male flowers are typically, but not always, smaller than female (van de Passe, 1605; Steven *et al.*, 2007). We therefore speculated that the smaller size of Firefly flowers, compared to the other double-flowered varieties, could reflect a sex-specific difference. The absence of stamens and carpels in the double flowers obscures visible differences between the sexes. Previous studies (Jenkins, 2002) identified a Y chromosome-specific marker that provides a PCR diagnostic for sex determination in *S. latifolia* and *S. dioica*. We used this PCR screen to determine the sex of the four double-flowered cultivars. This analysis reveals that Flore Pleno, Rosea Plena and Thelma Kay are female and Firefly is male (Fig. 3).

**Fig. 4.**
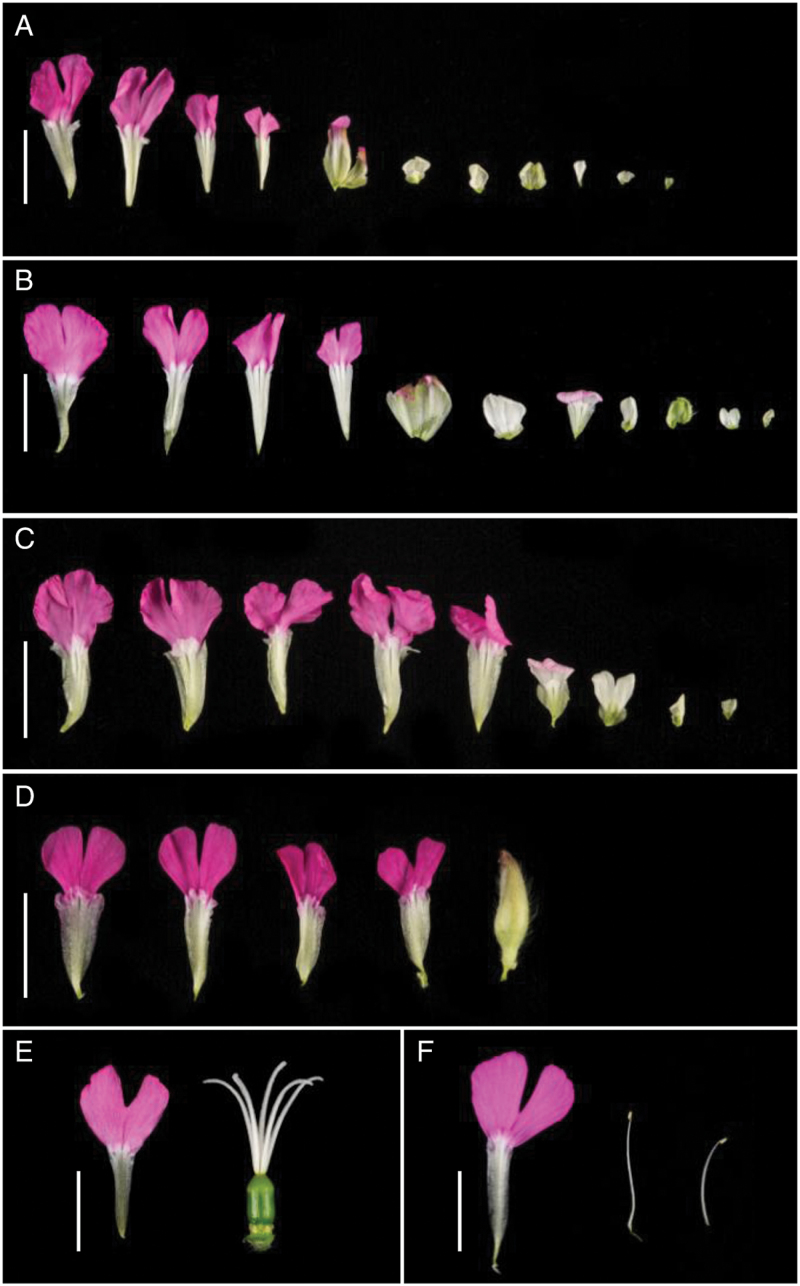
Dissection of individual flowers from: (A) Flore Pleno, (B) Rosea Plena, (C) Thelma Kay, (D) Firefly, (E) wild-type female, and (F) wild-type male. Every tenth petal from the dissected flower is shown in addition to other floral organs. Scale bars =1cm.

Flowers of the three female varieties have characteristic differences in petal colour; Rosea Plena is the palest and Thelma Kay the darkest pink. The flowers of Flore Pleno and Rosea Plena have two distinct groups of petals that form an inner and outer ring; in Thelma Kay there is no obvious boundary within the flower. In Rosea Plena the flower has good radial symmetry while in Flore Pleno the flower is less regular (Fig. 2). Dissection of flowers from all four varieties (Fig. 4) reveals more significant differences between them. The different floral architectures of Flore Pleno, Rosea Plena and Thelma Kay support the view that they are distinct rather than being one variety that has been renamed.

As shown in Fig. 4, Firefly has a flower structure most similar to that predicted by the simple ABC model of flower development, where stamens are converted to petals and carpels to sepals. Firefly flowers consist of outer sepals (not shown), a proliferation of petals similar in appearance to wild-type petals and a compact tube of sepals at the centre of the flower. This pattern is only found in Firefly and suggests that Firefly either represents a different mutant allele, mutation at a different locus, or a sex-specific difference in floral structure.

Unlike Firefly, petals in Flore Pleno, Rosea Plena and Thelma Kay are not consistent in colour or size and there are no central sepals. In all three cultivars the petals gradually decrease in size towards the centre of the flower, ranging from outer petals similar in size to wild-type (~15mm), to inner ones 1–2mm in length. The petals can also be divided into two groups by colour. The majority of petals are pink, as in wild-type, but towards the centre of the flower the petals fade to white with a mix of pink and white petals at the boundary. There is no distinct boundary at which this change occurs: it appears to reflect petal size (Fig. 4).

To further elucidate difference between the varieties we dissected and counted petals from 10 flowers of each variety. Thelma Kay flowers have a mean petal number of 101.7 (±3.6); the mean petal number in Flore Pleno and Rosea Plena are 92.2 (±5.2) and 91.5 (±4.1) respectively (Table 1); Thelma Kay lacks the additional level of concentric organisation seen in Flore Pleno and Rosea Plena. In these latter two varieties, the petals are divided into three distinct zones by the presence of two rings of fused petaloid sepals. The petaloid sepals can be seen in Fig. 4 as the fifth and eighth organ from the left in Flore Pleno and the fifth and ninth organ from the left in Rosea Plena. The base of the structure is green, veined and thicker than petal tissue, resembling normal sepals, but the top of the structure forms a ‘frill’ of thinner pink or white petal tissue. The rings are created from five of these structures fused together, much like a calyx within the flower, indicating that the 5-fold symmetry of the wild-type flowers has been retained. The spacing of these divisions is consistent between the two varieties (Table 1). The outermost ring occurs close to the boundary between pink and white petals, while the inner ring, less visible in the whole flower, is located approximately half way through the zone of white petals. These sepaloid petals give rise to the visible inner and outer rings in the intact flower shown in Fig. 2 and also evident in the image in Fig. 1.

The three female varieties have similar average numbers of pink and white petals, with a range across the varieties of 51.7–61.4 and 34.7–40.5 respectively (Table 1). But as described above, Thelma Kay consistently showed 10% more petals than Flore Pleno and Rosea Plena (Table 1). In contrast, Firefly flowers have an average of only 33.3 (±1.5) petals and although the flowers occasionally contain white petals, these occur infrequently and inconsistently. It is perhaps surprising that Thelma Kay shows such differences in floral phenotype from Flore Pleno and Rosea Plena given the documented origin of Thelma Kay as a variegated sport within an established population of double-flowered *S. dioica* (Brown, 1996).

### Comparison of genomic similarity within the four varieties

**Fig. 5.**
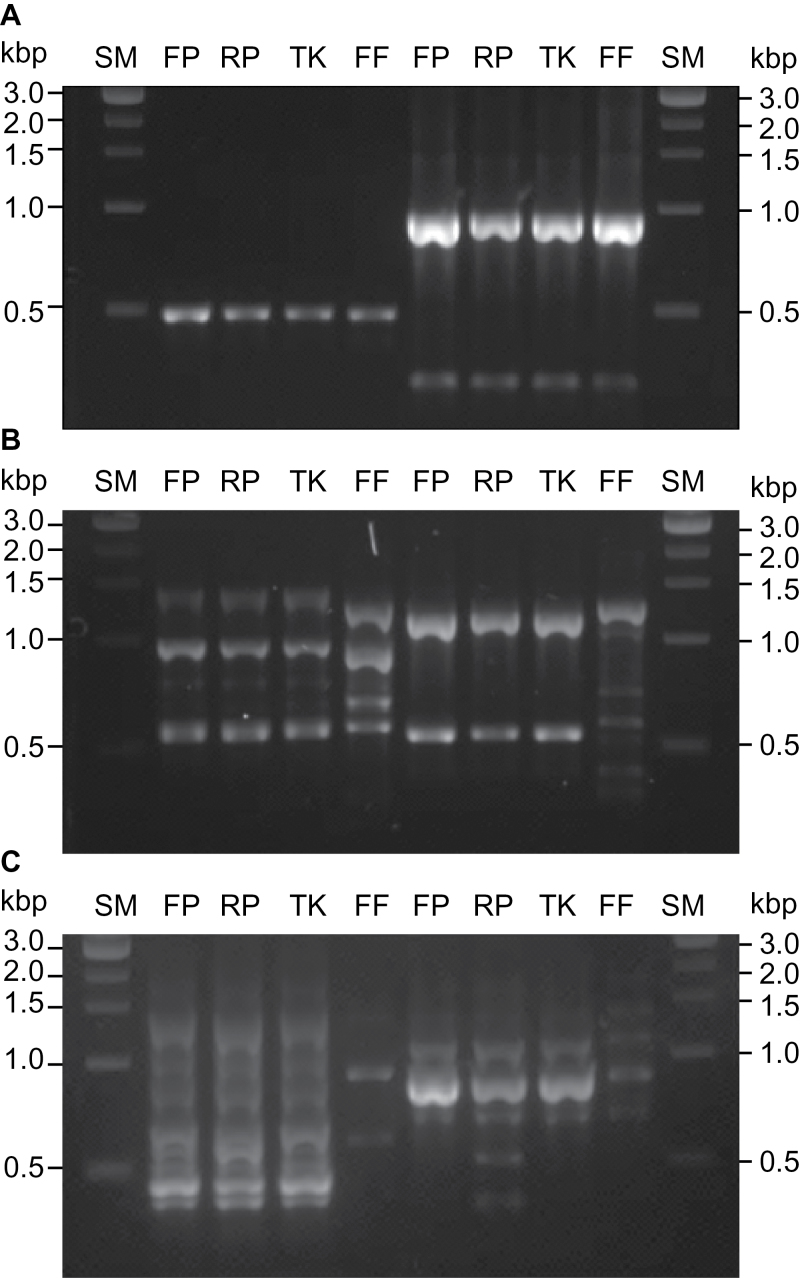
Representative genomic RAPD profile categories: (A) identical profiles in all varieties; (B) profiles shared by Flore Pleno, Rosea Plena and Thelma Kay; (C) profiles unique to Rosea Plena with distinguishing amplification products highlighted by asterisks. Each example shows data from two random primers. Varieties are indicated as: Flore Pleno (FP), Rosea Plena (RP), Thelma Kay (TK) and Firefly (FF). Size markers (SM) in kb.

To further investigate the relationship between the three female double varieties we used Random Amplification of Polymorphic DNA (RAPD) analysis to compare their genomic profiles alongside that of Firefly. We anticipated that, as a variegated sport arising from an existing variety, the RAPD profile of Thelma Kay would be more similar to either Flore Pleno or Rosea Plena depending on which variety was the origin of Thelma Kay. We used 20 different random primers for the analysis and this produced amplification profiles that fell into three categories. Fig. 5 shows representative results from two primers in each category. Six primers gave identical amplification profiles for all varieties (Fig. 5A), 11 primers produced identical amplification profiles in all three female varieties with a different profile in Firefly (Fig. 5B), reinforcing the suggestion that Firefly is of independent origin, and two primers amplified profiles unique to Rosea Plena (Fig. 5C). These data however reveal genetic similarities between all three female double varieties as well as phenotypic similarities.

### Prediction and characterisation of the locus responsible for double-flowered *Silene dioica*

**Fig. 6.**
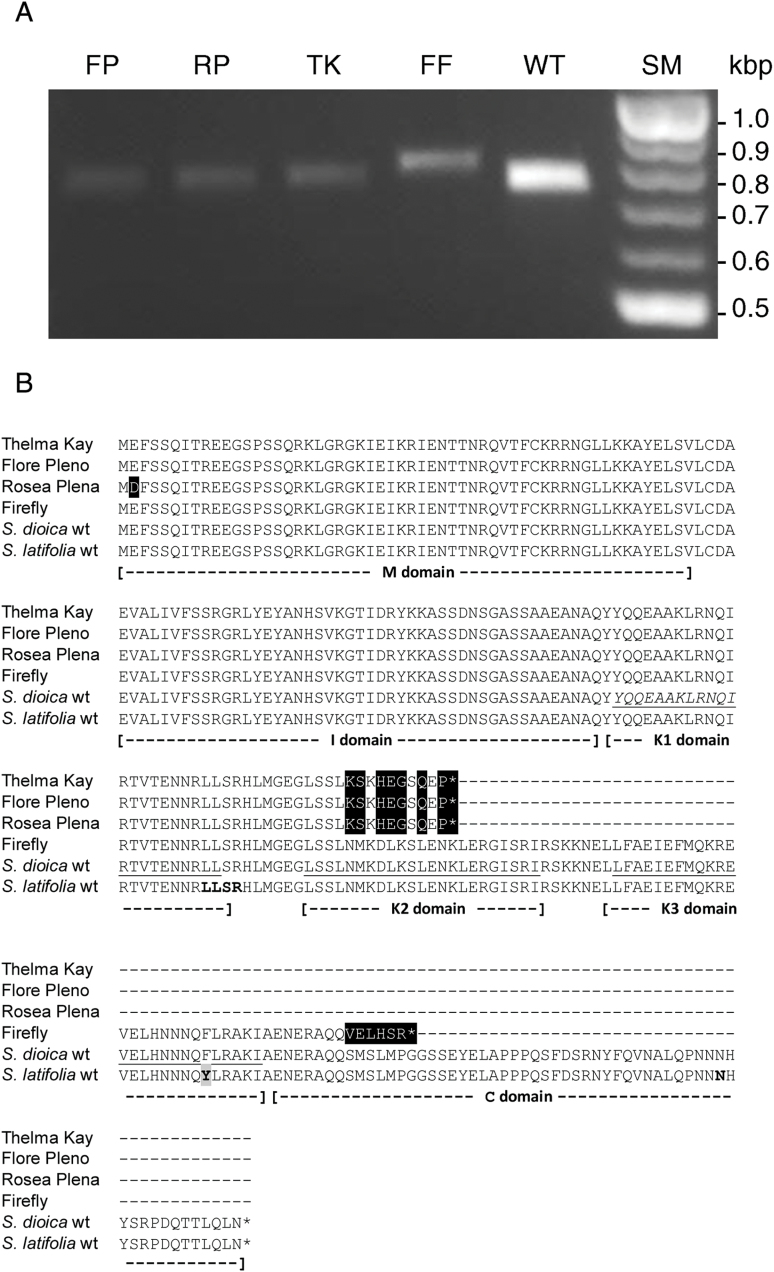
Analysis of the *SDM1* gene products in double-flowered varieties. (A) Agarose gel image of PCR amplification products of *SDM1* from flower bud cDNA of Flore Pleno (FP), Rosea Plena (RP), Thelma Kay (TK), Firefly (FF) and wild-type male (WT). Size markers (SM) in kb. (B) Alignment of predicted amino acid sequences of SDM1 in double-flowered varieties and wild-type *S. dioica* with *S. latifolia* SLM1 using the standard single letter code. Amino acid differences between SLM1 characterized here and the original SLM1 sequence (Hardenack *et al.*, 1994) are highlighted in bold. The single amino acid difference between SDM1 and SLM1 is shown in bold boxed gray. Stop codons are shown by an asterisk and divergence from wild-type is highlighted grey. Genbank accession numbers for *SDM1* sequences: male *Silene dioica* wild-type, KM598332; Flore Pleno, KM598329; Rosea Plena, KM598330; Thelma Kay, KM598331; Firefly, KM598328; *S. latifolia SLM1*: KP954655.

Previous studies (Hardenack *et al.*, 1994) identified *S. latifolia MADS1* (*SLM1*) as a putative C-function MADS-box gene based on homology to the *PLENA* gene in *Antirrhinum*, and *in situ* expression analysis revealed an expression profile consistent with typical C-function gene expression in the third and fourth floral whorls. We therefore investigated the expression and organization of the corresponding *S. dioica MADS1* gene (*SDM1*) as a potential candidate for the locus responsible for the double-flowered phenotype. The *SDM1* cDNA was amplified from wild-type male *S. dioica* and the four double varieties using primers designed from the first methionine to the stop codon of the open reading frame of the *S. latifolia* gene (Hardenack *et al.*, 1994). PCR products were obtained from all four double-flowered mutants indicating that the *SDM1* locus is transcribed in each variety. The PCR products were analysed by agarose gel electrophoresis (Fig. 6A). Products derived from Flore Pleno, Rosea Plena and Thelma Kay appear similar in size to wild-type. The product obtained from Firefly was visibly larger (Fig. 6A).

The *SDM1* PCR products were sequenced; all contain an extra 12bp and four conserved base substitutions when compared to the original *S. latifolia SLM1* sequence (Hardenack *et al.*, 1994). We noted that the missing 12bp in *SLM1* resulted in the loss of four amino-acid residues in the K1 domain of the encoded protein as compared to *SDM1*; this deletion was at the boundary of intron three and exon four. We then amplified by PCR the cDNA and corresponding genomic region of *SLM1* from our experimental population of *S. latifolia* and found that these 12 bases were present in our *S. latifolia* sequence (Supplementary Fig. S1). The original *SLM1* cDNA may therefore represent an alternate splice variant; we also noted four allelic base changes between the two *SLM1* cDNAs, one of which results in an amino acid substitution, and a single nucleotide polymorphism in intron three (Supplementary Fig. S1). Fig. 6B shows a multiple sequence alignment of the five predicted SDM1 polypeptide sequences and our *S. latifolia* SLM1. Comparison of the five *S. dioica* cDNA sequences revealed that Firefly *SDM1* contains a unique 44bp insertion. *SDM1* from Flore Pleno, Rosea Plena and Thelma Kay all share an identical 7bp insertion, indicating that these three female varieties carry the same original mutant allele. The Rosea Plena cDNA also contains a unique single nucleotide substitution, which results in substitution of Glu2 to Asp2 with the N-terminal M domain (Fig. 6B). The 7bp insertion in *SDM1* in the female varieties introduces an in-frame stop codon shortly after the insertion site. The 44bp Firefly *SDM1* insertion also results in premature termination of the encoded protein as the insertion contains an in-frame stop codon. Amino acid changes in the predicted proteins and introduced stop codons are shown in Fig. 6B. Truncation of the encoded protein in Firefly results in the loss of the C domain of SDM1 while in the female double-flowered mutants, the protein also lacks half of the K domain (Fig. 6B).

We also investigated whether other C-function MADS-box genes might exist in the *S. dioica* genome; *SLM1* was the only C-function-like gene identified in *S. latifolia* by cDNA library screening (Hardenack *et al.*, 1994). Blast analysis of our *S. dioica* genome assembly only identified contigs containing *SDM1*, and provided no evidence of other C-function genes; similarly, analysis of flower RNA-Seq data only identified a single *SDM1*-related transcript assembly. Blast searches of the *Silene* SiESTa database (Blavet *et al.*, 2011), and a *S. latifolia* reference genome sequence, also reveal only a single sequence alignment for *SLM1* and no other related C-function genes (Alex Widmer, personal communication).

### Comparison of the *SLM1* genomic DNA sequences in double-flowered mutants

**Fig. 7.**
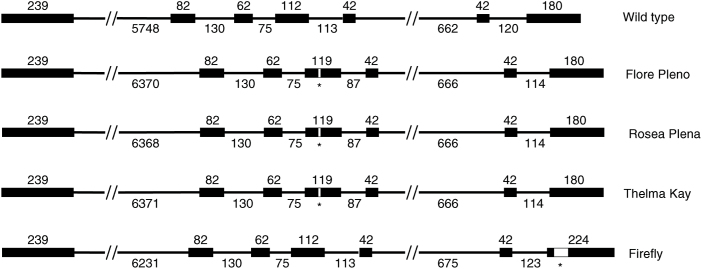
Schematic diagram of the genomic structure of the *SDM1* locus. The intron-exon structure of the *SDM1* locus is indicated for the four double-flowered varieties and wild-type *S. dioica*. Black boxes denote exons, lines denote introns, long introns are shown interrupted by //. The length of exons and introns in bp are shown above and below the genes respectively. White boxes marked * indicate the locations of insertion mutations that cause the mutant phenotypes.

To isolate the genomic sequence corresponding to each of the five *SDM1* cDNA clones we used available Illumina paired-end reads of genomic DNA sequence to assemble a highly fragmented draft genome assembly. This was used to identify contigs corresponding to the *SDM1* locus. These sequences helped inform primer design to amplify the full genomic region between the ATG and stop codon of the transcription unit for *SDM1*. Fig. 7 shows the structure of the *SDM1* locus in each of the four double varieties and wild-type *S. dioica.* The coding region is split into seven exons within either a 7.6kb wild-type gene or an 8.2kb gene in the double-flowered varieties. This analysis reveals that the 44bp insertion in Firefly *SDM1* is in exon seven while the 7bp insertion in the other three *SDM1* loci is within the fourth exon (Fig. 7).

Genomic DNA sequences show large numbers of differences between the five alleles including single nucleotide polymorphisms (SNPs) and large deletions and insertions (INDELs) (Supplementary Fig. S1). The wild-type sequence contains variations in DNA sequence not found in the four double-flowered mutants which is not surprising as it come from an unrelated population. Over 100 SNPs and INDELs distinguish Firefly from both wild-type and from the three female double-flowered mutants. The three female varieties, Flore Pleno, Rosea Plena and Thelma Kay, share very high levels of sequence similarity with a total of eight SNPs across the 8.2kb genomic region. In a comparison of SNPs between Flore Pleno, Rosea Plena and Thelma Kay, three of the eight are unique to Thelma Kay, four to Rosea Plena and only one to Flore Pleno. Only 11 SNPs are found between the mutants and wild-type *SDM1* within the coding region, 10 of which represent synonymous changes (Supplementary Fig. S1).

## Discussion

As a first step towards defining the relationship between the four available double-flowered varieties of *S. dioica* we established that Firefly, originally identified within a population of wild-type plants in 2009 (Blake, 2009), is male. The other three varieties, Flore Pleno, Rosea Plena and Thelma Kay, are female (Fig. 3). Firefly must therefore be of a distinct origin to the other mutants as the double-flowered mutants are sterile, and female mutants could not have promulgated a male line.

We know that Thelma Kay arose as a variegated sport (Brown, 1996), but it is not documented whether it was from a population of Flore Pleno or Rosea Plena. The relationship between Flore Pleno and Rosea Plena is undocumented. We considered three possible origins for Rosea Plena: (i) it segregated as a double-flowered mutant from the same original population of wild-type plants as Flore Pleno; (ii) it arose from a novel recessive mutation in an unrelated population; (iii) it originated as a somatic variant of Flore Pleno, was recognized as different and became an established variety.

Given the 200 years between the original documentation of the two varieties, and their geographically different origins, we considered the first possibility to be unrealistic as the original mutant heterozygote is unlikely to have been maintained and propagated. In considering a recessive mutation in *S. dioica* it is important to recognize that it cannot self: homozygous recessive individuals can only arise by crosses between male and female heterozygotes. Given the similarities between the floral architecture of Flora Pleno and Rosea Plena we considered it probable that the mutants share a genetic common origin rather than having arisen independently. RAPD data similarly suggested a common genetic relationship between the three female mutants (Fig. 5).

We speculated that the *S. dioica* homologue (*SDM1*) of the *S. latifolia* gene *SLM1*, would be responsible for the *S. dioica* double-flowered phenotypes. *SLM1* was identified by homology to the *Antirrhinum majus PLENA* gene and shows expression (Hardenack *et al.*, 1994) consistent with C-function MADS-box genes from other species such as *Arabidopsis thaliana* (Yanofsky *et al.*, 1990) and *Antirrhinum majus* (Bradley *et al.*, 1993). Mutation of *SLM1* would therefore be predicted to lead to a double-flowered phenotype. *SDM1* and *SLM1* cDNA sequences share over 99% nucleotide identity (Supplementary Fig. S1), the encoded proteins differ by only one amino-acid residue (Fig. 6B), and we have no evidence from available genomic and RNA-Seq data for additional C-function-like genes in *S. dioica* or *S latifolia.* These observations, together with the identification of two independent mutant alleles of *SDM1* that are each associated with the double-flowered mutant phenotype lead us to predict that *SDM1* is the locus responsible for the double-flowered mutants. However, the sterility of these mutants precludes classical genetic segregation or complementation analysis.

We were able to isolate cDNA sequences for *SDM1* from all four double-flowered mutants, which revealed that the locus was still expressed. Non-quantitative PCR analysis (Fig. 6A) revealed differences in PCR band intensity between the mutants and wild-type and this could reflect differences in transcript abundance. Analysis of cDNA sequences revealed that all four double-flowered mutants of *S. dioica* carry mutations within the *SDM1* locus and we interpret this as the potential cause of the double-flowered mutant phenotypes. Sequence comparisons further reinforced the independent origin of Firefly, which carries a unique allele with a 44bp insertion in exon seven (Fig. 6A). Our data also demonstrated that Flore Pleno, Rosea Plena and Thelma Kay all carry the same 7bp insertion into exon four (Fig. 6A) and this confirms their common origin and the development of Rosea Plena as a somatic variant of Flore Pleno.

The *SDM1* mutation in Flore Pleno, Rosea Plena and Thelma Kay causes a frame-shift, which results in truncation of the encoded polypeptide within the K2 domain. The MADS-box protein K domain is involved in protein-protein interactions and heterodimer formation (Yang *et al.*, 2003; Yang and Jack, 2004; Kaufmann *et al.*, 2005). This mutation in *SDM1* would therefore be predicted to disrupt the ability of SDM1 to interact with partner proteins. The frame-shift mutation in Firefly *SDM1* occurs near the start of the C domain. The C domain has been shown to be involved in stabilization of protein complex formation and transcriptional activation (Cho *et al.*, 1999; Egea-Cortines *et al.*, 1999; Honma and Goto, 2001; Pelaz *et al.*, 2001). Again, this mutation would be predicated to disrupt function of the encoded protein. The Glu2-Asp2 substitution found in Rosea Plena, as compared to wild-type and other mutants, is within the MADS domain, which is involved in DNA binding specificity (Nurrish and Treisman, 1995; Riechmann *et al.*, 1996). This mutation is however not anticipated to interfere with protein function as these two amino acids are similar in structure and both contain negatively charged R groups.

**Fig. 8.**
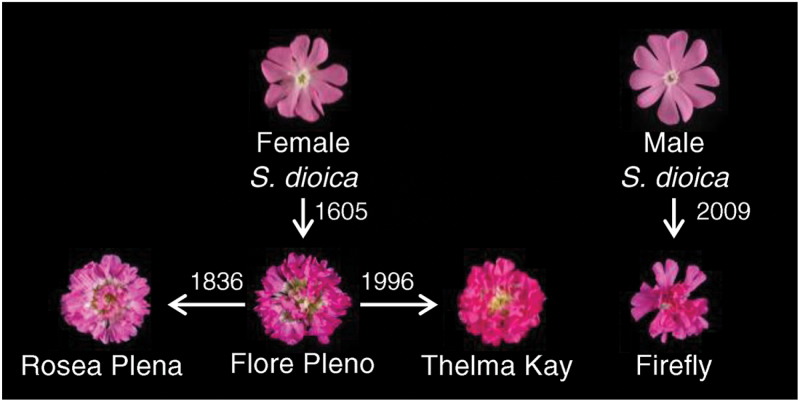
A model depicting the origins of the double-flowered varieties of *S. dioica.* Labelled photographs of flowers indicate varieties and arrows show descent based on genetic analysis. The date when the variety was first recorded is shown.

Comparison of the genomic sequences of *SDM1* (Supplementary Fig. S1) from Flore Pleno, Rosea Plena and Thelma Kay allows for comparison of individual differences between these varieties. When comparing the sequence of *SDM1* in Thelma Kay to that in Flore Pleno there are four SNPs, but when comparing Thelma Kay to Rosea Plena there are seven. The RAPD data (Fig. 5) shows that Thelma Kay and Flore Pleno have matching profiles while Rosea Plena occasionally differs. While it is not conclusive, our comparative analyses of *SDM1* sequences therefore suggest that Thelma Kay arose from Flore Pleno rather than from Rosea Plena. A schematic model for the predicted origins of the four double varieties is shown in Fig. 8.

When comparing the genomic sequence of *SDM1* in plants that do not share a common origin, the large numbers of accumulated SNPs and INDELs found are not unexpected given the different geographical origins of the plants. Within the three cultivars, Flore Pleno, Rosea Plena and Thelma Kay—which share a common origin—we identified SNPs at eight positions; these changes must reflect somatic variation that has accumulated since the reproductive isolation of the locus ~400 years ago, and may also reflect chimeric differences within each plant. It is therefore possible that somatic mutations either at the *SDM1* locus, or other loci, are responsible for the subtle differences in floral phenotypes, including colour. These double-flowered varieties of *S. dioica* provide a distinctive overview of phenotypic variation after 400 years of asexual reproduction in plants sharing the same original mutant allele.

The floral structure in Firefly is most similar to that predicted by the ABC model of flower development where stamens are replaced by petals and the carpel by a second whorl of sepals (Coen and Meyerowitz, 1991). As Firefly produces an average of 33.3 (±1.5) petals (Table 1), mutation of *SDM1* must also lead to an increase in organ primordia within the flower and not just conversion as seen in other double-flowered mutants of hermaphrodite species (Bowman *et al.*, 1989; Yanofsky *et al.*, 1990; Bradley *et al.*, 1993). The lower number of petals when compared to the three female mutants (Table 1) may reflect differences in the *SDM1* alleles but it is also possible that suppression of the central floral whorl by the Y chromosome limits the number of organs that can develop.

The presence of an inner whorl of sepals in Firefly suggests that the B-function MADS genes are not active in this central whorl while extended expression of B-function MADS-box genes into the centre of the flower would be predicted to create double flowers that produce repeated whorls of petals and no central sepals (Davies *et al.*, 1999), as found in the three female varieties. Previous studies showed no expression of B-function genes *SLM2* and *SLM3* in the fourth whorl of male *S. latifolia* flowers (Hardenack *et al.*, 1994). In contrast, low-level expression of *SLM2* and *SLM3* were detected in the fourth whorl of female flowers (Hardenack *et al.*, 1994). It is interesting to speculate that the floral phenotype of Firefly may be directly linked to the male-specific pattern of expression of MADS-box genes due to the dioecious nature of the species.

The possible causes of the difference in floral phenotypes between the female varieties and Firefly also include the difference in length of the truncated SDM1 protein and the genetic background. The presence of the complete SDM1 K domain in Firefly may allow some residual function, particularly in maintaining floral determinacy, although this would have to be through protein-protein interactions rather than modulating transcription because the K domain promotes dimerization rather than transcriptional activity (Yang *et al.*, 2003). While we cannot rule out the effect of either genetic background or protein function, we speculate that the difference in phenotype between Firefly and the female mutants could arise from the sex-specific differences in B-function MADS-box gene expression.

The phenotype in the female varieties is similar to that created by mutation of *PLENA* in *Antirrhinum* *majus* and *AGAMOUS* in *Arabidopsis thaliana,* where there is conversion of stamens to petals and the initiation of a new flower in place of the fourth whorl, leading to further proliferation of sepal tissue and multiple whorls of petals (Yanofsky *et al.*, 1990; Bradley *et al.*, 1993; Davies *et al.*, 1999). The three female varieties of *S. dioica* contain a similar proliferation of multiple whorls of petals and, in Flore Pleno and Rosea Plena, the rings of petaloid sepals could be seen to be equivalent to the reoccurring rings of sepals found in *plena* and *agamous* mutants although the occurrence of these rings does not form a regular repeat and sepal development is partial rather than being the initiation of a distinct flower.

In Thelma Kay the two internal rings of petaloid sepals have been lost (Fig. 4). Average petal number in Thelma Kay is slightly higher but total organ number is very similar (Table 1) so the absence of the petaloid sepals may represent a transformation to petal tissue. Thelma Kay flowers are closer to the phenotype seen in a *plena/farinelli* double mutant of *Antirrhinum majus*, which shows similar proliferation of petals and loss of the intervening whorls of sepals (Davies *et al.*, 1999), and *agamous superman/flo* double mutants in *Arabidopsis thaliana* (Schultz *et al.*, 1991; Bowman *et al.*, 1992). *SDM1* is the only C-function gene to be identified so far in *S. dioica*, but it is possible that an additional somatic mutation at a different locus of similar function to *FARINELLI* (Davies *et al.*, 1999) or *SUPERMAN* (Bowman *et al.*, 1992; Schultz *et al.*, 1991) is the cause of changes to floral phenotype in Thelma Kay.

In the 4th Century bc Theophrastus commented on the existence of roses with 100 petals (Hort, 1980). Whatever the basis for the changes that underpin the differences between Thelma Kay and its progenitor Flore Pleno, it is certainly a match for Theophrastus’ roses in terms of petal number.

## Supplementary data

Supplementary data can be found at *JXB* online.

Supplementary Fig. S1. Multiple sequence alignment of *SDM1* genomic DNA from different double-flowered varieties.

Supplementary Table S1. Petal and sepal counts, mean values and standard errors for individual flowers of double-flowered mutants.

Supplementary Data
