# Supplementary material for: Molecular characterisation of four double-flowered mutants of Silene dioica representing four centuries of variation
Source: J Exp Bot. 2015 Apr 15;66(11):3297–307. doi: 10.1093/jxb/erv139 (PMC4449544; doi:10.1093/jxb/erv139)
Supplement: Supplementary Data [file supp_66_11_3297__index.html]

Molecular characterisation of four double-flowered mutants of Silene dioica representing four centuries of variation — Molecular characterisation of four double-flowered mutants of Silene dioica representing four centuries of variation — Supplementary Data 

# Molecular characterisation of four double-flowered mutants of *Silene dioica* representing four centuries of variation

## Supplementary Data

Data files

**Files in this Data Supplement:**

- Supplementary Data - Supplementary Data
