# Supplementary material for: Molecular characterisation of four double-flowered mutants of Silene dioica representing four centuries of variation
Source: J Exp Bot. 2015 Apr 15;66(11):3297–307. doi: 10.1093/jxb/erv139 (PMC4449544; doi:10.1093/jxb/erv139)
Supplement: Supplementary Data [file supp_erv139_jexbot141861_file001.pdf]

Title: Molecular characterisation of four double-flowered mutants of Silene dioica representing four centuries of variation

Author: Elizabeth Ingle and Philip Gilmartin

Table S1 Petal and sepal counts, mean values and standard errors for individual flowers of *double-flower* mutants

Flore Pleno

Flower number	After outer sepals		Middle set of petals		Inner set of petals		Total petals		
	Pink	White	Pink	White	Pink	White	Pink	White	Total
1	44	6	0	13	0	11	44	30	74
2	49	0	2	23	0	15	51	38	89
3	48	9	7	17	0	19	55	45	100
4	49	0	16	5	0	10	65	15	80
5	43	0	0	70	0	15	43	85	128
6	52	0	1	20	0	22	53	42	95
7	42	1	1	14	0	24	43	39	82
8	38	5	1	22	0	14	39	41	80
9	59	0	15	13	0	22	74	35	109
10	49	0	1	14	0	21	50	35	85
Total	47	2	4	21	0	17	52	41	92
SE	1.9	1	2	5.7	0	1.6	3.4	5.6	5.2

Firefly

Flower number	After outer sepals		Middle set of petals		Inner set of petals		Total petals		
	Pink	White	Pink	White	Pink	White	Pink	White	Total
1	39	2	n/a	n/a	n/a	n/a	39	2	41
2	29	0	n/a	n/a	n/a	n/a	29	0	29
3	37	0	n/a	n/a	n/a	n/a	37	0	37
4	31	1	n/a	n/a	n/a	n/a	31	1	32
5	38	3	n/a	n/a	n/a	n/a	38	3	41
6	30	0	n/a	n/a	n/a	n/a	30	0	30
7	29	0	n/a	n/a	n/a	n/a	29	0	29
8	29	0	n/a	n/a	n/a	n/a	29	0	29
9	32	1	n/a	n/a	n/a	n/a	32	1	33
10	32	0	n/a	n/a	n/a	n/a	32	0	32
Total	33	1	n/a	n/a	n/a	n/a	33	1	33
SE	1.2	0.3	n/a	n/a	n/a	n/a	1.2	0.3	1.5

Thelma Kay

Flower number	After outer sepals		Middle set of petals		Inner set of petals		Total petals		
	Pink	White	Pink	White	Pink	White	Pink	White	Total
1	39	42	n/a	n/a	n/a	n/a	39	42	81
2	89	28	n/a	n/a	n/a	n/a	89	28	117
3	68	41	n/a	n/a	n/a	n/a	68	41	109
4	55	54	n/a	n/a	n/a	n/a	55	54	109
5	73	30	n/a	n/a	n/a	n/a	73	30	103
6	46	38	n/a	n/a	n/a	n/a	46	38	84
7	62	46	n/a	n/a	n/a	n/a	62	46	108
8	75	32	n/a	n/a	n/a	n/a	75	32	107
9	58	45	n/a	n/a	n/a	n/a	58	45	103
10	49	47	n/a	n/a	n/a	n/a	49	47	96
Total	61	40	n/a	n/a	n/a	n/a	61	40	102
SE	4.8	2.6	n/a	n/a	n/a	n/a	4.8	2.6	3.6

Rosea Plena

Flower number	After outer sepals		Middle set of petals		Inner set of petals		Total petals		
	Pink	White	Pink	White	Pink	White	Pink	White	Total
1	49	10	0	12	0	28	49	50	99
2	45	5	0	18	0	22	45	45	90
3	53	6	0	20	0	26	53	52	105
4	55	0	17	9	0	21	72	30	102
5	50	0	12	18	0	23	62	41	103
6	53	1	13	14	0	19	66	34	100
7	47	3	0	17	0	12	47	32	79
8	58	0	14	10	0	11	72	21	93
9	45	0	8	10	0	6	53	16	69
10	48	0	1	14	0	12	49	26	75
Total	50	3	7	14	0	18	57	35	92
SE	1.4	1.1	2.2	1.2	0	2.3	3.3	3.8	4.1

Figure S1 Multiple sequence alignment of SDM1 genomic DNA from different double-flowered varieties.

Genomic DNA sequence of SLM1 in Rosea plena (RP), Thelma Kay (TK), Flore pleno (FP), Firefly (FF) and wild type Silene dioica (SD) were aligned by Clustal Omega. Gaps are indicated by -, nucleotide identity by *. Exons are shown in bold type. Sequence polymorphisms unique to wild type are highlighted in yellow; those unique to Firefly are highlighted in blue and those common to Flore Pleno, Rosea Plena and Thelma Kay are highlighted in green. Single nucleotide polymorphisms that distinguish between Flore Pleno, Rosea Plena and Thelma Kay are highlighted in magenta. Insertion mutations which disrupt the amino acid sequence of the predicted protein are highlighted within red boxes. S. latifolia (SL) sequences covering exons and the region spanning the four amino acid deletion in SLM1, as compared to SDM1, are also included. Single nucleotide polymorphisms specific to SL are highlighted in red and the additional bases, as compared to the original SLM1 cDNA (Hardenack et al., 1994), at the junction of intron three and exon four are highlighted in grey.

```
FP      ATGGAGTTTTCAAGCCAAATTACAAGGGAAGAAGGTTCACCATCAAGCCAAAGGAAATTA    60
RP      ATGGATTTTTCAAGCCAAATTACAAGGGAAGAAGGTTCACCATCAAGCCAAAGGAAATTA    60
TK      ATGGAGTTTTCAAGCCAAATTACAAGGGAAGAAGGTTCACCATCAAGCCAAAGGAAATTA    60
FF      ATGGAGTTTTCAAGCCAAATTACAAGGGAAGAAGGCTCACCATCAAGCCAAAGGAAATTA    60
SD      ATGGAGTTTTCAAGCCAAATTACAAGGGAAGAAGGCTCACCATCAAGCCAAAGGAAATTA    60
        ***** ***************************** ************************

SL      ATGGAGTTTTCAAGCCAAATTACAAGGGAAGAAGGCTCACCATCAAGCCAAAGGAAATTA

FP      GGGAGAGGTAAAATCGAGATTAAGCGTATCGAAAACACGACAAATCGTCAAGTTACCTTC    120
RP      GGGAGAGGTAAAATCGAGATTAAGCGTATCGAAAACACGACAAATCGTCAAGTTACCTTC    120
TK      GGGAGAGGTAAAATCGAGATTAAGCGTATCGAAAACACGACAAATCGTCAAGTTACCTTC    120
FF      GGGAGAGGTAAAATCGAGATTAAGCGTATCGAAAACACGACAAATCGTCAAGTTACCTTC    120
SD      GGGAGAGGTAAAATCGAGATTAAGCGTATCGAAAACACGACAAATCGTCAAGTTACCTTC    120
        ************************************************************

SL      GGGAGAGGTAAAATCGAGATTAAGCGTATCGAAAACACGACAAATCGTCAAGTTACCTTC

FP      TGTAAGAGGAGAAATGGATTGCTTAAGAAAGCCTATGAATTATCTGTACTTTGTGATGCT    180
RP      TGTAAGAGGAGAAATGGATTGCTTAAGAAAGCCTATGAATTATCTGTACTTTGTGATGCT    180
TK      TGTAAGAGGAGAAATGGATTGCTTAAGAAAGCCTATGAATTATCTGTACTTTGTGATGCT    180
FF      TGTAAGAGGAGAAATGGATTGCTTAAGAAAGCCTATGAATTATCTGTACTTTGTGATGCT    180
SD      TGTAAGAGGAGAAATGGATTGCTTAAGAAAGCCTATGAATTATCTGTACTTTGTGATGCT    180
        ************************************************************

SL      TGTAAGAGGAGAAATGGATTGCTTAAGAAAGCCTATGAATTATCTGTACTTTGTGATGCT

FP      GAAGTTGCCCTTATTGTTTTCTCAAGCCGCGGCCGTCTCTATGAATATGCTAATCATAGG    240
RP      GAAGTTGCCCTTATTGTTTTCTCAAGCCGCGGCCGTCTCTATGAATATGCTAATCATAGG    240
TK      GAAGTTGCCCTTATTGTTTTCTCAAGCCGCGGCCGTCTCTATGAATATGCTAATCATAGG    240
FF      GAAGTTGCCCTTATTGTTTTCTCAAGCCGCGGCCGTCTCTATGAATATGCTAATCATAGG    240
SD      GAAGTTGCCCTTATTGTTTTCTCAAGCCGCGGCCGTCTCTATGAATATGCTAATCATAGG    240
        ************************************************************

SL      GAAGTTGCCCTTATTGTTTTCTCAAGCCGCGGCCGTCTCTATGAATATGCTAATCATAG

FP      TA--------TATCTTATACTTTCTCCGTCTTAATCATTTGTTTATATATTATATTCTGT    292
RP      TA--------TATCTTATACTTTCTCCGTCTTAATCATTTGTTTATATATTATATTCTGT    292
TK      TA--------TATCTTATACTTTCTCCGTCTTAATCATTTGTTTATATATTATATTCTGT    292
FF      TA--------TATCTTATACTTTCTCCGTCTTAATCATTTGTTTATATATTATATTCTGT    292
SD      TATATGACTATATCTTATACTTTCTCCGTCTTAATCATTTGTTTATATATTATATTTTGT    300
        **        ********************************************** ***

FP      GTTATGGGTATTTTAATTAAAGGCGGACAAATGATTGAAATGAAGTTTCACACATTTTTG    352
RP      GTTATGGGTATTTTAATTAAAGGCGGACAAATGATTGAAATGAAGTTTCACACATTTTTG    352
TK      GTTATGGGTATTTTAATTAAAGGCGGACAAATGATTGAAATGAAGTTTCACACATTTTTG    352
FF      GTTATGGGTATTTTAATTAAAGGCGGACAAATGATTGAAA-CAGGTTTCACACATTTTTG    351
SD      GTTATGGGTATTTTAATTAAAGGCGGACAAATGATTGAAATGAAGTTTCACACATTTTTG    360
        ****************************************  * ****************

FP      ATGTTAAAA-GTGGCTAATTTGGGTTGGATTTAAAAAATGGGATGAATTAATTAAGTTTT    411
RP      ATGTTAAAA-GTGGCTAATTTGGGTTGGATTTAAAAAATGGGATGAATTAATTAAGTTTT    411
TK      ATGTTAAAA-GTGGCTAATTTGGGTTGGATTTAAAAAATGGGATGAATTAATTAAGTTTT    411
FF      ATGTTAAAA-GTGGCTAATTTGGGTTGGATTTAAAAAATGGGATGAATTAATTAAGTTTT    410
SD      ATGTTAAAAAGTGGCTAATTTGGGTTGGATTTAAAAAATGGGATGAATTAATTAAGTTTT    420
        ********* **************************************************

FP      AAGAATTATTGAATTTTTTAATGAAATAAATAAAGAATTAAAATTGTCTGATTTTGTTAC    471
RP      AAGAATTATTGAATTTTTTAATGAAATAAATAAAGAATTAAAATTGTCTGATTTTGTTAC    471
TK      AAGAATTATTGAATTTTTTAATGAAATAAATAAAGAATTAAAATTGTCTGATTTTGTTAC    471
FF      AAGAATTATTGAATTTTTTAATGAAATAAATAAAGAATTAAAA-TGTCTGATTTTGTTAC    469
SD      AAGAATTATTGAATTTTTTAATGAAATAAATAAAGAATTAAAATTGTCTGATTTTGTTAC    480
        ******************************************* ****************

FP      ATTTTTGTTTTTTTCTAAGTAAAAGCTTAAAAAAGCTATTTTACTATTT-TTTATTTTAG    530
RP      ATTTTTGTTTTTTTCTAAGTAAAAGCTTAAAAAAGCTATTTTACTATTT-TTTATTTTAG    530
TK      ATTTTTGTTTTTTTCTAAGTAAAAGCTTAAAAAAGCTATTTTACTATTT-TTTATTTTAG    530
FF      ATTTTTGTTTTTTTCTAAGTAAAAGCTTAAAAAAGCTATTTTACTATTTTTTTATTTTAG    529
SD      ATTTTTGTTTTTTTCTAAGTAAAAGCTTAAAAAAGCTATTTTACTATTTTTTTATTTTAG    540
        ************************************************* **********
```

```
FP      TTGTTTAGATTGCTAGAAACATAGATCTAGTTTGTAATAGATGGAAATATATAGTAGGAG      590
RP      TTGTTTAGATTGCTAGAAACATAGATCTAGTTTGTAATAGATGGAAATATATAGTAGGAG      590
TK      TTGTTTAGATTGCTAGAAACATAGATCTAGTTTGTAATAGATGGAAATATATAGTAGGAG      590
FF      TTGTTTAGATTGCTAGAAACATAGATCTAGTTTGTAATAGATGGAAATAAATAGTAGGAG      589
SD      TTGTTTAGATTGCTAGAAACATAGATCTAGTTTGTAATAGATGGAAATAAATAGTAGGAG      600
        ************************************************* **********

FP      TAAGTAAGCCTAATTTCTTGAATTGTACTTAGATTTTCAGACTAAAGAAGAAATAAAAGT      650
RP      TAAGTAAGCCTAATTTCTTGAATTGTACTTAGATTTTCAGACTAAAGAAGAAATAAAAGT      650
TK      TAAGTAAGCCTAATTTCTTGAATTGTACTTAGATTTTCAGACTAAAGAAGAAATAAAAGT      650
FF      TAAGTAAGCCTAATTTCTTGAATTGTACTTAGATTTTCAGACTAAAGAAGAAATAAAAGT      649
SD      TAAGTAAGCCTAATTTCTTGAATTGTACTTAGATTTTCAGACTAAAGAAGAAATAAAAGT      660
        ************************************************************

FP      TAAGAAACAACAACATATATAGAATAGGCATATAAAATAATCTGATTGAGTTTTTGGGAA      710
RP      TAAGAAACAACAACATATATAGAATAGGCATATAAAATAATCTGATTGAGTTTTTGGGAA      710
TK      TAAGAAACAACAACATATATAGAATAGGCATATAAAATAATCTGATTGAGTTTTTGGGAA      710
FF      TAAGAAACAACAACATATATAGAATAGGCATATAAAATAATCTGATTGAATTTTTGGGAA      709
SD      TAAGAAACAACAACATATATAGAATAGGCATATAAAATAATCTGATTGAGTTTTTGGGAA      720
        ************************************************* **********

FP      AGTTTTATTTCAGGTCAGTGAGATCAGCATCCCTAAATTCCCCCTCTCCATTGTTGCAA      770
RP      AGTTTTATTTCAGGTCAGTGAGATCAGCATCCCTAAATTCCCCCTCTCCATTGTTGCAA      770
TK      AGTTTTATTTCAGGTCAGTGAGATCAGCATCCCTAAATTCCCCCTCTCCATTGTTGCAA      770
FF      AGTTTTATTTCAGGTCAGTGAGATCAGCATCCCTAAATTCCTCCCTCTCCATTGTTGCAA      769
SD      AGTTTTATTTCAGGTCAGTGAGATCAGCATCCCTAAATTCCTCCCTCTCCATTGTTGTAA      780
        ***************************************** *************** **

FP      AAAAAAAAAAATA------TATATTTCTCCTTTTTCTCGATTTTACAAATTTGTATTCGA      824
RP      AAAAAAAAAAATA------TATATTTCTCCTTTTTCTCGATTTTACAAATTTGTATTCGA      824
TK      AAAAAAAAAAATA------TATATTTCTCCTTTTTCTCGATTTTACAAATTTGTATTCGA      824
FF      AAAAAATAAAAAAAAA----ATATTTCTCCTTTTTCTCGATTTTACAAATTTGTATTGGA      825
SD      AAAAAAAAAAAATAAAAAAAATATTTCTCCTTTTTCTCGATTTTACAAAATTGTATTGGA      840
        ****** **** *       ***************************** ******* **

FP      TTGTTAGTTTCTGATGTTTCTGTAGATGTCGTATGACTATTAATCACATTTTTACAAAAT      884
RP      TTGTTAGTTTCTGATGTTTCTGTAGATGTCGTATGACTATTAATCACATTTTTACAAAAT      884
TK      TTGTTAGTTTCTGATGTTTCTGTAGATGTCGTATGACTATTAATCACATTTTTACAAAAT      884
FF      TTGTTAGTTTCTGATGTTTCTGTAGATGTCGTATGACTATTAATCACATTTTTATAAAAT      885
SD      TTGTTAGTTTCTGATGTTTCTGTAGATGTCGTATGACTATTAATCACATTTTTATAAAAT      900
        ****************************************************** *****

FP      TATATTCGGGTTGTTTCTGATGGTTCTGTAGATGC---GCGACGATTTTTCACATTTT--      939
RP      TATATTCGGGTTGTTTCTGATGGTTCTGTAGATGC---GCGACGATTTTTCACATTTT--      939
TK      TATATTCGGGTTGTTTCTGATGGTTCTGTAGATGC---GCGACGATTTTTCACATTTT--      939
FF      TGTATTCGGATTGTTTCTGATGGTTCTGTAGATGCCGTGTGACGATTTTTCACATTTT--      943
SD      TGTATTCGGGTTGTTTCTGATGGTTCTGTAGATGCCGTGTGACGATTTTTCACATTTTTT      960
        * ******* *************************   * ******************

FP      CTTAAAAGAAAGAATTAGTCATTATTTTACAAATTTTTATTCGGGTTGTTTCTGATGGTT      999
RP      CTTAAAAGAAAGAATTAGTCATTATTTTACAAATTTTTATTCGGGTTGTTTCTGATGGTT      999
TK      CTTAAAAGAAAGAATTAGTCATTATTTTACAAATTTTTATTCGGGTTGTTTCTGATGGTT      999
FF      CTTAAAGGAAAGAAT--GTCATTATTTTACAAATTTTTATTCGGGTTGTTTCTGATGGTT      1001
SD      CTTAAAAGAAAGAAT--GTCATTATTTTACAAATTTTTATTCGGGTTGTTTCTGATGGTT      1018
        ****** ********  *******************************************

FP      CTGTAGATGTTGTATGACGATTTTTCACATTTTATTTAA-GAAAGCATTATTATTCAT--      1056
RP      CTGTAGATGTTGTATGACGATTTTTCACATTTTATTTAA-GAAAGCATTATTATTCAT--      1056
TK      CTGTAGATGTTGTATGACGATTTTTCACATTTTATTTAA-GAAAGCATTATTATTCAT--      1056
FF      CTGTAGATGTTGTATGACGATTTTTCACATTTTATTTAAAGAAATCATTATTATTCATCG      1061
SD      CTGTAGATGTTGTATGACGATTTTTCACATTTTATTTAAAGAAATCATTATTATTCATCG      1078
        *************************************** **** *************
```

```
FP      --------AAATTTGTATTCGGGTTGTTTCTGATGGTTCTGTAGATGTCGTAAGACGATT      1108
RP      --------AAATTTGTATTCGGGTTGTTTCTGATGGTTCTGTAGATGTCGTAAGACGATT      1108
TK      --------AAATTTGTATTCGGGTTGTTTCTGATGGTTCTGTAGATGTCGTAAGACGATT      1108
FF      ATTTTGACAAATTTGTATTCGGGTTGTTTCTGATGGTTCTGTAGATGTCGTAAGACGATT      1121
SD      ATTTTGACAAATTTGTATTCGGGTTGTTTCTGATGGTTCTGTAGATGTCGTAAGACGATT      1138
                ****************************************************

FP      TTCACAATTTATTCATGGAAGCATTATTATTCATCTCTAGTACATGCCCATGATCTATGA      1168
RP      TTCACAATTTATTCATGGAAGCATTATTATTCATCTCTAGTACATGCCCATGATCTATGA      1168
TK      TTCACAATTTATTCATGGAAGCATTATTATTCATCTCTAGTACATGCCCATGATCTATGA      1168
FF      TTCACAATTTATTCATGGAAGCATTATTATTCATCTCTAGTACATGCCCATGATCTATGA      1181
SD      TTCACATTTTATTCATGGAAGCATTATTATTCATCTCTAGTACATGCCCATGATCTATGA      1196
        ****** *****************************************************

FP      TGCTATGCTAGATGATGAGGGTGATGGGGTTGGTTACCCATAAAGATAAAACAAAAGGAC      1228
RP      TGCTATGCTAGATGATGAGGGTGATGGGGTTGGTTACCCATAAAGATAAAACAAAAGGAC      1228
TK      TGCTATGCTAGATGATGAGGGTGATGGGGTTGGTTACCCATAAAGATAAAACAAAAGGAC      1228
FF      TGCTATGCTAGATGATGAGGGTGATGGGGTTGGTTACCCATAAAGATAAAACAAAA-GAC      1240
SD      TGCTATGCTAGATGATGAGGGTGATGGGGTTGGTTACCCATAAAGATAAAACAAAAGGAC      1258
        ******************************************************** ***

FP      ACCCATTAAAGTTTGGTTAAAACACCCATCATATACCAATTCTAAATTACAACTAACTAA      1288
RP      ACCCATTAAAGTTTGGTTAAAACACCCATCATATACCAATTCTAAATTACAACTAACTAA      1288
TK      ACCCATTAAAGTTTGGTTAAAACACCCATCATATACCAATTCTAAATTACAACTAACTAA      1288
FF      ACCCATTAAAGTTTGGTTAAAACACCCATCATATACCAACTCTAAATTACAACT----AA      1296
SD      ACCCATTAAAGTTTGGTTAAAACACCCATCATATACCAACTCTAAATTACAACT----AA      1314
        *************************************** **************    **

FP      TTGTAGTAGATATATATAGGGCTCACCCTAAAATAATTAATTTTCAGTTTTCCTAACATT      1348
RP      TTGTAGTAGATATATATAGGGCTCACCCTAAAATAATTAATTTTCAGTTTTCCTAACATT      1348
TK      TTGTAGTAGATATATATAGGGCTCACCCTAAAATAATTAATTTTCAGTTTTCCTAACATT      1348
FF      TTGTAGTAGATATATATAGGGCTCACCCTAAAATAATTAATTTTCAATTTTCCTAACATT      1356
SD      TTGTAGTAGATATATATAGGGCTCACCCTAAAATAATTAATTTTCAGTTTTCCTAACATT      1374
        ********************************************** *************

FP      TTT-TTTTTGCAATTTCATAACCTTAATTAGGGTTTAATATGGTCATACACAACTTAATT      1407
RP      TTT-TTTTTGCAATTTCATAACCTTAATTAGGGTTTAATATGGTCATACACAACTTAATT      1407
TK      TTT-TTTTTGCAATTTCATAACCTTAATTAGGGTTTAATATGGTCATACACAACTTAATT      1407
FF      TT--TTTTTGCAATTTCATAACCTTAATTAGGGTTTAATATGGTCATACACAACTTAATT      1414
SD      TTTTTTTTTGCAATTTCATAACCTTAATTAGGGTTTAATATGGTCATACACAACTTAATT      1434
        **  ********************************************************

FP      TTCTTAACCCATAATTAGTTAACCCTTCAACTTGGTGTTAAGTATGATAGATACTAATTT      1467
RP      TTCTTAACCCATAATTAGTTAACCCTTCAACTTGGTGTTAAGTATGATAGATACTAATTT      1467
TK      TTCTTAACCCATAATTAGTTAACCCTTCAACTTGGTGTTAAGTATGATAGATACTAATTT      1467
FF      TTCTTAACCCATAATTAGTTAACCCTTCAACTTGGTGTTAAGTATGATAGATACTAATTT      1474
SD      TTCTTAACCCATAATTAGTTAACCCTTCAACTTGGTGTTAAGTATGATAGATACTAATTT      1494
        ************************************************************

FP      GGTCAAAAACTAACCAAAATTAGGGCTTATAATACTTCTATTAATATAAAATGGTAATGT      1527
RP      GGTCAAAAACTAACCAAAATTAGGGCTTATAATACTTCTATTAATATAAAATGGTAATGT      1527
TK      GGTCAAAAACTAACCAAAATTAGGGCTTATAATACTTCTATTAATATAAAATGGTAATGT      1527
FF      GGTCAAAAACTAACCAAAATTAGGGCTTATTAAACTTCTATTAATATAAAATGGTAATGT      1534
SD      GGTCAAAAACTAACCAAAATTAGGGCTTATTAAACTTCTATTAATATAAAATGGTAATGT      1554
        ****************************** * **************************

FP      TCAAATTTATTGAAAAATACTCAAATTTAGGTAAGTTTGTCAATTTCAGTAACATTATAG      1587
RP      TCAAATTTATTGAAAAATACTCAAATTTAGGTAAGTTTGTCAATTTCAGTAACATTATAG      1587
TK      TCAAATTTATTGAAAAATACTCAAATTTAGGTAAGTTTGTCAATTTCAGTAACATTATAG      1587
FF      TCAAATTTATTGAAAAATACTCAAATTTAGGTAAGTTTGTCAATTTCAGTAACATTATAG      1594
SD      TCAAATTTATTGAAAAATACTCAAATTTAGGTAAGTTTGTCAATTTCAGTAACATTATAG      1614
        ************************************************************
```

```
FP      CTAGTAGTTACAGCACATACACTCCAACAACAACAGGTTTCCTAGATTTTGCTTTTCTTA      1647
RP      CTAGTAGTTACAGCACATACACTCCAACAACAACAGGTTTCCTAGATTTTGCTTTTCTTA      1647
TK      CTAGTAGTTACAGCACATACACTCCAACAACAACAGGTTTCCTAGATTTTGCTTTTCTTA      1647
FF      CTAGTAGTTACAGCATATACACTCCAACAACAACAGGTTTCCTAGATTTTGCTTTTCTTA      1654
SD      CTAGTAGTTACAGCACATACACTCCAACAACAACAGGTTTCCTAGATTTTGCTTTTCTTA      1674
        *************** ********************************************

FP      CTTTAGCATTTTTT-TTTCAATTTATTTAAAACTTTTTTTCCTTGTTTCCCTAATTAGGG      1706
RP      CTTTAGCATTTTTT-TTTCAATTTATTTAAAACTTTTTTTCCTTGTTTCCCTAATTAGGG      1706
TK      CTTTAGCATTTTTT-TTTCAATTTATTTAAAACTTTTTTTCCTTGTTTCCCTAATTAGGG      1706
FF      CTTTAGCATTTTTTTTTTCAATTTATTTAAAACTTTTTTTCCTTGTTTCCCTAATTAGGG      1714
SD      CTTTAGCATTTTTT-TTTCAATTTATTTAAAACTTTTTTTCCTTGTTTCCCTAATTAGGG      1733
        ************** *********************************************

FP      CTTACACATAGCTACACCGAGACTGTAAGATGGTCTTATTCTATAATTTAAGTCTTTTCC      1766
RP      CTTACACATAGCTACACCGAGACTGTAAGATGGTCTTATTCTATAATTTAAGTCTTTTCC      1766
TK      CTTACACATAGCTACACCGAGACTGTAAGATGGTCTTATTCTATAATTTAAGTCTTTTCC      1766
FF      CTTACACATAGCTACACCGAGACTGTAAGATGGTCTTATTCTATAATTTACGTCTTTTCC      1774
SD      CTTACACATAGCTACACCGAGACTGTAAGATGGTCTTATTCTATAATTTACGTCTTTTCC      1793
        ************************************************** *********

FP      GAATTTTTTGGGTGTTGATGAAGTAAAGAGTTTTCTGACAATAAAGAAGTAAGAAAAGGG      1826
RP      GAATTTTTTGGGTGTTGATGAAGTAAAGAGTTTTCTGACAATAAAGAAGTAAGAAAAGGG      1826
TK      GAATTTTTTGGGTGTTGATGAAGTAAAGAGTTTTCTGACAATAAAGAAGTAAGAAAAGGG      1826
FF      GAATTTTTTGGGTGTTGATGAAGTAAAGAGTTTTCTGACAATAAAGAAGTAAGAAAAGGG      1834
SD      GAATTTTTTGGGTGTTGATGAAGTAAAGAGTTTTCTGACAATAAAGAAGTAAGAAAAGGG      1853
        ************************************************************

FP      TATGATCGACCAATGTTAAAAGAATCGTCTTAGACTGTAAGACTGGCTTATTCTATAATT      1886
RP      TATGATCGACCAATGTTAAAAGAATCGTCTTAGACTGTAAGACTGGCTTATTCTATAATT      1886
TK      TATGATCGACCAATGTTAAAAGAATCGTCTTAGACTGTAAGACTGGCTTATTCTATAATT      1886
FF      TATGATCGACCAATGTTAAAAGAATCGTCTCAAGCTGTAAGACTGTCTTATTCTATAATT      1894
SD      TATGATCGACCAATGTTAAAAGAATCGTCTCAAGCTGTAAGACTGTCTTATTCTATAATT      1913
        ****************************** *  *********** **************

FP      TGTTTTTTCCGATATTTTGGGTGTTGATGAAGTAAGAAAAGGGCATAAGACGAATGTTAA      1946
RP      TGTTTTTTCCGATATTTTGGGTGTTGATGAAGTAAGAAAAGGGCATAAGACGAATGTTAA      1946
TK      TGTTTTTTCCGATATTTTGGGTGTTGATGAAGTAAGAAAAGGGCATAAGACGAATGTTAA      1946
FF      TATTTTTTCCGATATTTTGGGTGTTGATG--GTAAGAAAAGGGCATAAGACGAATGTTAA      1952
SD      TGTTTTTTCCGATATTTTGGGTGTTGATGAAGTAAGAAAAGGGCATAAGATGAATGTTCA      1973
        * ***************************  ******************* ******* *

FP      AAGAACCGTCTGAGACTGTAAGACGGTCTTATTCTATAATTTGCGTTTTTTTGATTTATT      2006
RP      AAGAACCGTCTGAGACTGTAAGACGGTCTTATTCTATAATTTGCGTTTTTTTGATTTATT      2006
TK      AAGAACCGTCTGAGACTGTAAGACGGTCTTATTCTATAATTTGCGTTTTTTTGATTTATT      2006
FF      AAGAACCGT-TGAGACTGTAAGACGGTCTTATTCTATAATTTGCGTTTTTTTGATTTATT      2011
SD      AAGAACCGGCTGAGACTGTAAGACGGTCTTATTCTATAATTTGCGTTTTTTTGATTTATT      2033
        ********  **************************************************

FP      TTGGGTGTTGATGAAGTAAGGAGTAAAAGGGTATGAGATGAATGTTACGAGAACAGTCTC      2066
RP      TTGGGTGTTGATGAAGTAAGGAGTAAAAGGGTATGAGATGAATGTTACGAGAACAGTCTC      2066
TK      TTGGGTGTTGATGAAGTAAGGAGTAAAAGGGTATGAGATGAATGTTACGAGAACAGTCTC      2066
FF      TTGGGTGTTGATGAAGTAAGGAGTAAAAGGGTATGAGATGAATGTTACCAGAACAGTCTC      2071
SD      TTGGGTGTTGATGAAGTAAGGAGTAAAAGGGTATGAGATGAATGGTACGAGAACAGTCTC      2093
        ******************************************** *** ***********

FP      AACAGACTATAAGATCGTCTTATTCTATAATTTGCGATATTTTCGAGTTTTCTGGGTGGT      2126
RP      AACAGACTATAAGATCGTCTTATTCTATAATTTGCGATATTTTCGAGTTTTCTGGGTGGT      2126
TK      AACAGACTATAAGATCGTCTTATTCTATAATTTGCGATATTTTCGAGTTTTCTGGGTGGT      2126
FF      AAGAGACTGTAAAACCGTCTTATTCTATAATTTGCGATATTTTTGAGTTTTTTGGGTGGT      2131
SD      AAGAGACTGTAAAACCGTCTTATTCTATAATTTGCGATATTTTCGAGTTTTTTGGGTGGT      2153
        ** ***** *** * **************************** ******* ********
```

```
FP      GATGAGGTAAGGAGAATTCTGACAAGAAAGAAGTAAGAAAGGGGTAGTGATATGATTAAA      2186
RP      GATGAGGTAAGGAGAATTCTGACAAGAAAGAAGTAAGAAAGGGGTAGTGATATGATTAAA      2186
TK      GATGAGGTAAGGAGAATTCTGACAAGAAAGAAGTAAGAAAGGGGTAGTGATATGATTAAA      2186
FF      GATGAAGTAAGGAGAATTCTGACAAGAAAGAAGTAAGAAAGGGGTAGTGATATGATTAAA      2191
SD      GATGAAGTAAGGAGAATTCTGACAAGAAAGAAGTAAGAAAGGGGTAGTGATATGATTAAA      2213
        ***** ******************************************************

FP      TTTCCAAGAGTTAGGGTAAAGTCTTTTAACTGCAATAGTGAAAGAAAAATAAAATACATA      2246
RP      TTTCCAAGAGTTAGGGTAAAGTCTTTTAACTGCAATAGTGAAAGAAAAATAAAATACATA      2246
TK      TTTCCAAGAGTTAGGGTAAAGTCTTTTAACTGCAATAGTGAAAGAAAAATAAAATACATA      2246
FF      TTTCCAAGAGTTAGGGTAAAGTCTTTTAACTGCAATAGTGAAAGAAAAATAAAATACATA      2251
SD      TTTCCAAGAGTTAGGGTAAAGTCTTTTAACTGCAATAGTGAAAGAAAAATAAAATACATA      2273
        ************************************************************

FP      AGAGTTATAGTAACTCACTTAGATTGTGAAAGGATCTAACTTTATTTTTTCTTTCATAAT      2306
RP      AGAGTTATAGTAACTCACTTAGATTGTGAAAGGATCTAACTTTATTTTTTCTTTCATAAT      2306
TK      AGAGTTATAGTAACTCACTTAGATTGTGAAAGGATCTAACTTTATTTTTTCTTTCATAAT      2306
FF      AGAGTTATAGTAACTCACTTAGATTGTGAAAGGATCTAACTTTATTTTTTCTTTCATAAT      2311
SD      AGAGTTATAGTAACTCACTTAGATTGTGAAAGGATCTAACTTTATTTTTTCTTTCATAAT      2333
        ************************************************************

FP      AATAATAAATTTTTACACAAAACTTTGATCTTTTCAATTTTCAATTTAAAGAACAAGTCT      2366
RP      AATAATAAATTTTTACACAAAACTTTGATCTTTTCAATTTTCAATTTAAAGAACAAGTCT      2366
TK      AATAATAAATTTTTACACAAAACTTTGATCTTTTCAATTTTCAATTTAAAGAACAAGTCT      2366
FF      AATAATAAATTTTTACACAAAACTTTGATCTTTT-------CAATTTAAAGAACAAGTCT      2364
SD      AATAATAAATTTTTACACAAAACTTTGATCTTTT-------CAATTTAAAGAACAAGTCT      2383
        **********************************       *******************

FP      TTCTCTTTGTGATCTCATCTTTGGTTGTGTGCTTAAATTGCAAAAACCAACAAAATTTCT      2426
RP      TTCTCTTTGTGATCTCATCTTTGGTTGTGTGCTTAAATTGCAAAAACCAACAAAATTTCT      2426
TK      TTCTCTTTGTGATCTCATCTTTGGTTGTGTGCTTAAATTGCAAAAACCAACAAAATTTCT      2426
FF      TTCTCTTTGTGATCTCATCTTTGGTTGTGTGCTTAAATTGCAAAAACCAACAAAATTTCT      2424
SD      TTCTCTTTGTGATCTCATCTTTGGTTGTGTGCTTAAATTGCAAAAACCAACAAAATTTCT      2446
        ************************************************************

FP      ACTGCTGTTGTTTCAGTGATTAAGTTAAAGTGTGACATACAGTTGTTCATTTGTGTGTCT      2486
RP      ACTGCTGTTGTTTCAGTGATTAAGTTAAAGTGTGACATACAGTTGTTCATTTGTGTGTCT      2486
TK      ACTGCTGTTGTTTCAGTGATTAAGTTAAAGTGTGACATACAGTTGTTCATTTGTGTGTCT      2486
FF      ACTGCTGTTGTTTCAGTGATTAAGTTAAAGTGTGACATACAGTTGTTCATTTGTGTGTCT      2484
SD      ACTGCTGTTGTTTCAGTGATTAAGTTAAAGTGTGACATACAGTTGTTCATTTGTGTGTCT      2506
        ************************************************************

FP      ATGAATTCAAGTTACATCATCTTCTTTCACTTTATTAGGAAAAATTATTTAACTTTCTTG      2546
RP      ATGAATTCAAGTTACATCATCTTCTTTCACTTTATTAGGAAAAATTATTTAACTTTCTTG      2546
TK      ATGAATTCAAGTTACATCATCTTCTTTCACTTTATTAGGAAAAATTATTTAACTTTCTTG      2546
FF      ATGAATTCAAGTTACATCATCTTCTTTCACTTTATTAGGAAAAATTATTTAACTTTCTTG      2544
SD      ATGAATTCAAGTTACATCATCTTCTTTCACTTTATTAGGAAAAATTATTTAACTTTCTTG      2566
        ************************************************************

FP      GGACTAGTTAGATCTTGTATATATAGATATATTTTTACTCTTATAAATTTATTAAAAAAG      2606
RP      GGACTAGTTAGATCTTGTATATATAGATATATTTTTACTCTTATAAATTTATTAAAAAAG      2606
TK      GGACTAGTTAGATCTTGTATATATAGATATATTTTTACTCTTATAAATTTATTAAAAAAG      2606
FF      GGACTAGTTAGATCTTGTATATATAGATATATTTTTACTCTTATAAATTTATTAAAAAAG      2604
SD      GGACTAGTTAGATCTTGTATATATAGATATATTTTTACTCTTATAAATTTATTAAAAAAG      2626
        ************************************************************

FP      AAAAAAAAATTAACATGTACTATATATTAATAATATACTATCATATCATCTTTTATTAGG      2666
RP      AAAAAAAAATTAACATGTACTATATATTAATAATATACTATCATATCATCTTTTATTAGG      2666
TK      AAAAAAAAATTAACATGTACTATATATTAATAATATACTATCATATCATCTTTTATTAGG      2666
FF      AA-AAAAATTTAACATGTCCTATATATTAATAATATACTATCATATCATCTTTTATTAGG      2663
SD      AAAAAAAATTTAACATGTACTATATATTAATAACATACTAGCATATCATCTTTTATTAGG      2686
        ** ***** ********* ************** ****** *******************
```

```
FP          AAAGTGGTCCAGATTGTACCGATACCAGAACTGATCACTCTGAGTTTTCTCATTTAAAAT          2726
RP          AAAGTGGTCCAGATTGTACCGATACCAGAACTGATCACTCTGAGTTTTCTCATTTAAAAT          2726
TK          AAAGTGGTCCAGATTGTACCGATACCAGAACTGATCACTCTGAGTTTTCTCATTTAAAAT          2726
FF          AAAGTGGTCCAGATTGTACCGATACCAGAACTGATGACTCTGAGTTTTCTCATTTAAAAT          2723
SD          AAAGTGGTCCAGATTGTACCGATACCAGAACTGATGACTCTGAGTTTTCTCATTTAAAAT          2746
            *********************************** ************************

FP          TTCCCGGCCCACTTGGTTGGAAACTAGTTAGAAGTAAAGACAAATGATTTTACCTAAAGT          2786
RP          TTCCCGGCCCACTTGGTTGGAAACTAGTTAGAAGTAAAGACAAATGATTTTACCTAAAGT          2786
TK          TTCCCGGCCCACTTGGTTGGAAACTAGTTAGAAGTAAAGACAAATGATTTTACCTAAAGT          2786
FF          TTCCCGGCCCACTTGGTTGGAAACTAGTTAGAAGTAAAGACAAATGATTTTACCTAAAGT          2783
SD          TTCCCGGCCCACTTGGTTGGAAACTAGTTAGAAGTAAAGACAAATGATTTTACCTAAAGT          2806
            ************************************************************

FP          TTTATAGGTCGGACGAGACATTCCACTTTCTCGGACCCCATAACCTCAACTACATTATT-          2845
RP          TTTATAGGTCGGACGAGACATTCCACTTTCTCGGACCCCATAACCTCAACTACATTATT-          2845
TK          TTTATAGGTCGGACGAGACATTCCACTTTCTCGGACCCCATAACCTCAACTACATTATT-          2845
FF          TTTATAGGTCGGACGAGACATTCCACTTTCTCGGACCCCATAACCTCAACTACATTATTA          2843
SD          TTTATAGGTCGGACTAGACATTCCACACTCTCGGACCCCATAACCTCAATTACATTATTA          2866
            ************** ************  ******************** ********* 

FP          TTTCGAGTTAGGTTTACACCT--TTTTTTTCACGAGAAAAATCGCACAAGTTAATTAAAT          2903
RP          TTTCGAGTTAGGTTTACACCT--TTTTTTTCACGAGAAAAATCGCACAAGTTAATTAAAT          2903
TK          TTTCGAGTTAGGTTTACACCT--TTTTTTTCACGAGAAAAATCGCACAAGTTAATTAAAT          2903
FF          TTTCGAGTTAGGTTTACACCT--TTTTTTTCACAAGGAAAATCACTCAAGTTAATTAAAT          2901
SD          TTTCGAGTTAGGTTTACATCTTTTTTTTTTCACAAGGAAAATTGCGCAAGTTAATTAAAT          2926
            ****************** **  ********** ** *****  * **************

FP          CTAACGTAATCTAACCCGATTCAAATAGACCGAGATCGAGTTA-----------------          2946
RP          CTAATGTAATCTAACCCGATTCAAATAGACCGAGATCGAGTTA-----------------          2946
TK          CTAACGTAATCTAACCCGATTCAAATAGACCGAGATCGAGTTA-----------------          2946
FF          CTAACGTAATCTAACCCGATTCAAATCAACCGAGATCGAGTTAACTTTTATAGGTCTGAC          2961
SD          CAAACGTAATCTAACCCGATTCAAATCAACCGAGATCGAGTTAACTTTTATAGGTCTGAC          2986
            * ** *********************  ***************                 

FP          -------------CTCGAACCCATATTCCCAACTACATTATTAATTCGAGTTGGGTTTAG          2993
RP          -------------CTCGAACCCATATTCCCAACTACATTATTAATTCGAGTTGGGTTTAG          2993
TK          -------------CTCGAACCCATATTCCCAACTACATTATTAATTCGAGTTGGGTTTAG          2993
FF          AAGACATTCCACTCTCGAACCCATATTCTCAACTACATCATTGTTTTGAGTTAGGTTTAA          3021
SD          AAGACATTCCACTCTCGAACCCATATTCTCAACTACATCATTGTTTTGAGTTAGGTTTAA          3046
                         *************** ********* ***  ** ***** ****** 

FP          TTATTTTTACAAAGGGAAAAACCTCTCGAGTTAATTTAATCTAACGTAATCCAACCCGAT          3053
RP          TTATTTTTACAAAGGGAAAAACCTCTCGAGTTAATTTAATCTAACGTAATCCAACCCGAT          3053
TK          TTATTTTTACAAAGGGAAAAACCTCTCGAGTTAATTTAATCTAACGTAATCCAACCCGAT          3053
FF          TTATTTTTTCAAAGGGAAAAA-CCCTCGAGTTAATTTAATCTAACGTAATCTAACCCGAT          3080
SD          TTATTTTTTCAAAGGGAAAAAACCCTCGAGTTAATTTAATCTAACGTAATCTAACCCGAT          3106
            ******** ************ * ************************** *********

FP          TCAAATTGATCAAGATCGGAACAAGTAAAACCTTGAAATATGGAGAATTTAAGCTATGGA          3113
RP          TCAAATTGATCAAGATCGGAACAAGTAAAACCTTGAAATATGGAGAATTTAAGCTATGGA          3113
TK          TCAAATTGATCAAGATCGGAACAAGTAAAACCTTGAAATATGGAGAATTTAAGCTATGGA          3113
FF          TTAAATCGATCGAGATCGGAACAAGTAAAACCTTGAAATATGGAGAATTTAAGCTATGGA          3140
SD          TTAAATCGATCAAGATCGGAACAAGTAAAACCTTGAAATATGGAGAGTTTAAGCTATGGA          3166
            * **** **** ********************************** *************

FP          ATTAAAGTGTAGTCAGGGTTGTATAGGCTTGTAGCTTGGAGTATATGATAAAAATGGTGG          3173
RP          ATTAAAGTGTAGTCAGGGTTGTATAGGCTTGTAGCTTGGAGTATATGATAAAAATGGTGG          3173
TK          ATTAAAGTGTAGTCAGGGTTGTATAGGCTTGTAGCTTGGAGTATATGATAAAAATGGTGG          3173
FF          ATTAAAGTGTAGTCAGAGTTG--TAGGCTTGTAGCTTGGAGTATATGATAAAAATGGTGG          3198
SD          ATTAAAGTGTAGTCAGAGTTG--TAGGCTTGTAGCTTGGAGTATATGATAAAAATGGTGG          3224
            **************** ****  *************************************
```

```
FP      ATATTAGGGTAACCGACGGTTAATATACAAAGGGTAAAAAGGGGTGTTGACACGTGATGT      3233
RP      ATATTAGGGTAACCGACGGTTAATATACAAAGGGTAAAAAGGGGTGTTGACACGTGATGT      3233
TK      ATATTAGGGTAACCGACGGTTAATATACAAAGGGTAAAAAGGGGTGTTGACACGTGATGT      3233
FF      ATATTAGGGTAACCGACGGTTAATATACAAAGGGTAAAAAGGGTTGTTGACACGTGATGT      3258
SD      ATATTAGGGTAACCGACGGTTAATATACAAAGGGTAAAAAGGGGTGTTGACACGTGATGT      3284
        ******************************************* ****************

FP      GATCTTGACCGTTGATTTATTTCAAGCCAATCATATCACTGCAATACGTCGAGGAGATTA      3293
RP      GATCTTGACCGTTGATTTATTTCAAGCCAATCATATCACTGCAATACGTCGAGGAGATTA      3293
TK      GATCTTGACCGTTGATTTATTTCAAGCCAATCATATCACTGCAATACGTCGAGGAGATTA      3293
FF      GATCTTGACCGTTGATTTATTTCAAGCCAATCATATCACTGCAATACGTCGAGGAGATTA      3318
SD      GATCTTGACCGTTGATTTATTTCAAGCCAATCATATCACTGCAATACGTCGAGGAGATTA      3344
        ************************************************************

FP      AGGTATCACCAAAATCTAGGTGAACTAGGTAATATTATGTTCATTAAAATACAATAAAAT      3353
RP      AGGTATCACCAAAATCTAGGTGAACTAGGTAATATTATGTTCATTAAAATACAATAAAAT      3353
TK      AGGTATCACCAAAATCTAGGTGAACTAGGTAATATTATGTTCATTAAAATACAATAAAAT      3353
FF      AGGTATCACCAAAATCTAGGTGAACTAGGTAATATTATATTCATTAAAATACAATAAAAT      3378
SD      AGGTATCACCAAAATCTAGGTGAACTAGGTAATAAAATATTCATTAAAATACAATAAAAT      3404
        **********************************  ** *********************

FP      ATTAAAAAAAAAATGTAAAAAGTATAAAATTAAATTCTTTACTGTAGTTGTTGTCATTTCA      3413
RP      ATTAAAAAAAAAATGTAAAAAGTATAAAATTAAATTCTTTACTGTAGTTGTTGTCATTTCA      3413
TK      ATTAAAAAAAAAATGTAAAAAGTATAAAATTAAATTCTTTACTGTAGTTGTTGTCATTTCA      3413
FF      ATTAAAAAAAAAATGTAAAAAGTATAAAATTAAATTCTTTACTGTAGTTGTTGTCATTTCA      3438
SD      ATTAAAAAAAAAATGTAAAAAGTATAAAATTAAATTCTTTACTGTAGTTGTTGTCATTTCA      3464
        ************************************************************

FP      TACTAAATTATATCACGTCAGAAAAGAGTGAACCACAGTAAAAAAA-AATAAAGTTACTG      3472
RP      TACTAAATTATATCACGTCAGAAAAGAGTGAACCACAGTAAAAAAA-AATAAAGTTACTG      3472
TK      TACTAAATTATATCACGTCAGAAAAGAGTGAACCACAGTAAAAAAA-AATAAAGTTACTG      3472
FF      TACTAAATTATATCACGTCAGAAAAGAGTGAACCACAGTAAAAAAAAAAAAAAATTACTG      3498
SD      TACTAAATTATATCACGTCAGAAAAGAGTGAACCACAGTAACAAA--AAAAAAGTTACTG      3522
        ***************************************** ***  ** *** ******

FP      TATTGAAAAACTGAATTTTTTGAAAAAAACTGTAGCTTTTCGTGTAGGACTAAATTAAAT      3532
RP      TATTGAAAAACTGAATTTTTTGAAAAAAACTGTAGCTTTTCGTGTAGGACTAAATTAAAT      3532
TK      TATTGAAAAACTGAATTTTTTGAAAAAAACTGTAGCTTTTCGTGTAGGACTAAATTAAAT      3532
FF      TATTGAAAAACTGAATTTTTTGAAAAAAACTGTAGCTTTTCGTGTAGGACTAAATTAAAT      3558
SD      TATTGAAAAACTGAATTTTTTGAAAAAAACTGTAGCTTTTCGTGTAGGACTAAATTAAAT      3582
        ************************************************************

FP      TGATTAATGATTGATTTGACTGATTATAATTACTGGGTTTTTTGTTGGATTTCGTAGATT      3592
RP      TGATTAATGATTGATTTGACTGATTATAATTACTGGGTTTTTTGTTGGATTTCGTAGATT      3592
TK      TGATTAATGATTGATTTGACTGATTATAATTACTGGGTTTTTTGTTGGATTTCGTAGATT      3592
FF      TGATTAATGATTGATTTGACTGATTATAATTACTGGGTTTTTTGTTGGATTTCGTAGATT      3618
SD      TGATTAATGATTGATTTGACTGATTATAATTACTGGGTTTTTTGTTGGATTTCGTAGATT      3642
        ************************************************************

FP      TTAATGATGATTTTGATTTGGTACAAGAATAAATCAGAATTCATGATTTATTTATTTTAC      3652
RP      TTAATGATGATTTTGATTTGGTACAAGAATAAATCAGAATTCATGATTTATTTATTTTAC      3652
TK      TTAATGATGATTTTGATTTGGTACAAGAATAAATCAGAATTCATGATTTATTTATTTTAC      3652
FF      TTAATGATGATTTTGATTTGGTACAAGAATAAATCAGAATTCATGATTTATTTATTTTAC      3678
SD      TTAATGATGATTTTGATTTGGTACAAGAATAAATCAGAATTCATGATTTATTTATTTTAC      3702
        ************************************************************

FP      TGTTTGTTCTTACTTCTACTCAATCTAGTTTGAGTTATTTGTGGTACGGTAACTATTTTG      3712
RP      TGTTTGTTCTTACTTCTACTCAATCTAGTTTGAGTTATTTGTGGTACGGTAACTATTTTG      3712
TK      TGTTTGTTCTTACTTCTACTCAATCTAGTTTGAGTTATTTGTGGTACGGTAACTATTTTG      3712
FF      TGTTTGTTCTTACTTCTACTCAATCTAGTTTGAGTTATTTGTGGTACGGTAACTATTTTG      3738
SD      TGTTTGTTCTTACTTCTACTCAATCTAGTTTGAGTTATTTGTGGTACGGTAACTATTTTA      3762
        ***********************************************************
```

```
FP      GGTATCTTTCTAATGATTTACCATGCATCTTATTTTTAACTTATAGATTATACGTGTTTT      3772
RP      GGTATCTTTCTAATGATTTACCATGCATCTTATTTTTAACTTATAGATTATACGTGTTTT      3772
TK      GGTATCTTTCTAATGATTTACCATGCATCTTATTTTTAACTTATAGATTATACGTGTTTT      3772
FF      GGTATCTTTCTAATGATTTACCATTCATCTTATTTTTAACTTATAGATTATACGTGTTTT      3798
SD      GGTATCTTTCTAATGATTTACCATGCATCTTATTTTTAACTTATAGATTATACGTGTATT      3822
        ************************ ******************************** **

FP      TTTGATAAGTTTTATTTTATTTACCTATGTGTAATTCACTAGACTATGATGTTTTTCCTA      3832
RP      TTTGATAAGTTTTATTTTATTTACCTATGTGTAATTCACTAGACTATGATGTTTTTCCTA      3832
TK      TTTGATAAGTTTTATTTTATTTACCTATGTGTAATTCACTAGACTATGATGTTTTTCCTA      3832
FF      TTTGATAAGTTTTATTTTATTTACCTATGTGTAATTCACTTGACTATGATGTTTTTCCTA      3858
SD      TTTGTTAAGTTTTATTTTATTTACCTATGTGTAATTCACTAGACTATGATGTTTTTCCTA      3882
        **** *********************************** *******************

FP      TATTTTGTTAACTAAGTGTCTTTTAAGATGGTACTATCCGTCTTAAATTTAAGACTAGTC      3892
RP      TATTTTGTTAACTAAGTGTCTTTTAAGATGGTACTATCCGTCTTAAATTTAAGACTAGTC      3892
TK      TATTTTGTTAACTAAGTGTCTTTTAAGATGGTACTATCCGTCTTAAATTTAAGACTAGTC      3892
FF      TATTTTGTTAACTAAGTGTCTTTTAAGATGGTACTATCCGTCTTAAATTTAAGACTAGTC      3918
SD      TATTTTGTTAACTAAGTGTCTTTTAAGATGGTACTATCCGTCTTAAATTTAAGACTAGTC      3942
        ************************************************************

FP      AAGTGCATACCTCTTGTA-------TATATAAGAATGCATATGAGAAAA-TAATATATTT      3944
RP      AAGTGCATACCTCTTGTA-------TATATAAGAATGCATATGAGAAAA-TAATATATTT      3944
TK      AAGTGCATACCTCTTGTA-------TATATAAGAATGCATATGAGAAAA-TAATATATTT      3944
FF      AAGTACA-CCCCATTGTATACGGAGTATATAAGAATGCATATGAGAAAAATAATATATT-      3976
SD      AAGTGCATACCCCTTGTATAC-------ATAAGAATGCATATGAGAAAA-TAATATATTT      3994
        **** **  **  *****          ********************* *********

FP      GTC-TTATATATATAAGTGA-------TATGATAATTGACCTGTCTTAAAGTTTAAGATG      3996
RP      GTC-TTATATATATAAGTGA-------TATGATAATTGACCTGTCTTAAAGTTTAAGATG      3996
TK      GTC-TTATATATATAAGTGA-------TATGATAATTGACCTGTCTTAAAGTTTAAGATG      3996
FF      GTCTTTATATATATACGTGATACGGAGTATGATAGTTGACCTGTTTTAAAGTTTAAGATA      4036
SD      GTC-TTATATATATAAGTGA-------TATGATAGTTGACCTGTCTTAAAGTTTAAGATG      4046
        *** *********** ****       ******* ********* **************

FP      GATAGTGCCGTCTTAAGAATTTGTGTATTTTATTTTGGATGTTTTTGTCTATAAATGTCT      4056
RP      GATAGTGCCGTCTTAAGAATTTGTGTATTTTATTTTGGATGTTTTTGTCTATAAATGTCT      4056
TK      GATAGTGCCGTCTTAAGAATTTGTGTATTTTATTTTGGATGTTTTTGTCTATAAATGTCT      4056
FF      GATAGTGCCATTTTAAGAATTTGTGTA---------------------------------      4063
SD      GATAGTGTCGTCTTAAGAATTTGTGTATTTTATTTTGGATGTTTTTGTCTATAAATGTCT      4106
        ******* * * ***************

FP      TAAATTTTAGCTGCTTTAGAGAACAATATGAATGTAGTTTCATCAATAGAAAATTGTATA      4116
RP      TAAATTTTAGCTGCTTTAGAGAACAATATGAATGTAGTTTCATCAATAGAAAATTGTATA      4116
TK      TAAATCTTAGCTGCTTTAGAGAACAATATGAATGTAGTTTCATCAATAGAAAATTGTATA      4116
FF      --------------------------------------------------------TATC      4067
SD      TAAATCTTAGCTGCTTTAGAGAACAATATGAATGTAGTTTCATCAATAGAAAATTGTATA      4166
                                                                ***

FP      TCAAAATATTGTATTTTATTTGAAACGCGCC-ATCTTAATTGA--TTTTTTTTTTTTTGT      4173
RP      TCAAAATATTGTATTTTATTTGAAACGCGCC-ATCTTAATTGA--TTTTTTTTTTTTTGT      4173
TK      TCAAAATATTGTATTTTATTTGAAACGCGCC-ATCTTAATTGA--TTTTTTTTTTTTTGT      4173
FF      ATAATTTATTTTATTTTATTTGAAACGCGCCCATCATAATTGATTTTTTTTTTTTTTTGT      4127
SD      TCAAAATATTGTATTTTATTTGAAACGCGCC-ATCTTAATTGA-TTTTTTTTTTTTTTGT      4224
          **  **** ******************** *** *******  ***************

FP      GAAACGATTTTAAACAAAAATAATATAAAACTACAAAACCGTGATATCTGTATCTCTAAA      4233
RP      GAAACGATTTTAAACAAAAATAATATAAAACTACAAAACCGTGATATCTGTATCTCTAAA      4233
TK      GAAACGATTTTAAACAAAAATAATATAAAACTACAAAACCGTGATATCTGTATCTCTAAA      4233
FF      AAAACGATTTTAAACAAAAATAATATAAAACTACAAAACTGTGATATCTGTATCTCTAAA      4187
SD      GAAACGATTTTAAACAAAAATAATATAAAACTACAAAACCGTGATATCTGTATCTCTAAA      4284
         ************************************* *********************
```

```
FP      ATAATTTAAGACGAGATTTTTTT--TCCTTTTTTGAGGTAAATTTATGACGAGGAATTTT      4291
RP      ATAATTTAAGACGAGATTTTTTT--TCCTTTTTTGAGGTAAATTTATGACGAGGAATTTT      4291
TK      ATAATTTAAGACGAGATTTTTTT--TCCTTTTTTGAGGTAAATTTATGACGAGGAATTTT      4291
FF      ATAATTTAAGACGATATTTTTTTTTTCCTTTGTTGAGGTAAATTTATGACGAGGGATTTA      4247
SD      ATAATTTAAGATGAGATTTTTTTTTTCCTTTTTTGAGGTAAATTTATGACGAGGAATTTT      4344
        *********** ** ********  ****** ********************** ****

FP      AA------TTTTTTTTTTAAAGAATATGTAATTTCACACTAGTGCAAAATAGGCTATATG      4345
RP      AA------TTTTTTTTTTAAAGAATATGTAATTTCACACTAGTGCAAAATAGGCTATATG      4345
TK      AA------TTTTTTTTTTAAAGAATATGTAATTTCACACTAGTGCAAAATAGGCTATATG      4345
FF      AAAAAATATATTTTTGTTTAAGAATATGTAATTTCACACTAGTGCAAAATAGGCTTTATG      4307
SD      AATTTTTTTTTTTTTTTTAAAGAATATGTAATTTCACACTAGTGCAAAATAGGCTTTATG      4404
        **      * ***** ** ************************************ ****

FP      TAAGTATTTGCAATTACTTATAATTTTGTTCAAGAAATGAGCTTATGTATGGTATTGAAC      4405
RP      TAAGTATTTGCAATTACTTATAATTTTGTTCAAGAAATGAGCTTATGTATGGTATTGAAC      4405
TK      TAAGTATTTGCAATTACTTATAATTTTGTTCAAGAAATGAGCTTATGTATGGTATTGAAC      4405
FF      TAAGTATTTGCAATTACTTATAATTTTGTACAAGAAATGAGCTTATGTATGGTATTGAAC      4367
SD      CAAGTATTTGCAATTACTTATAATTTTGTTCAAGAAATGAGCTTATGTATGGTATTGAAC      4464
         **************************** ******************************

FP      TTAGTATCATTATTATGTTAATTATCCACAATAGAA------------------------      4441
RP      TTAGTATCATTATTATGTTAATTATCCACAATAGAA------------------------      4441
TK      TTAGTATCATTATTATGTTAATTATCCACAATAGAA------------------------      4441
FF      TTAGTATCATTATTATGTTAATTATCCACAATATAATTTTGTACAAGAAATGAGCTTATG      4427
SD      TTAGTATCATTATTATGTTAATTATCCACAATAGAA------------------------      4500
        ********************************* **

FP      TATGACATTTACATTCATTTTAGTAATAAGGTGACATTGAAACTCGACTTGTAGTACCAC      4501
RP      TATGACATTTACATTCATTTTAGTAATAAGGTGACATTGAAACTCGACTTGTAGTACCAC      4501
TK      TATGACATTTACATTCATTTTAGTAATAAGGTGACATTGAAACTCGACTTGTAGTACCAC      4501
FF      TATGACATTTACATTCATTTTAGTAATAAGGTGACATTGAAACTCGACTTGTAGTACCAC      4487
SD      TATGACATTTACATTCATTTTAGTAATAAGGTGACATTGAAACTCGACTTGTAATACCAC      4560
        ***************************************************** ******

FP      CGGTTAGTACTCTATCACTAGACATAATAATACCCAATTCTTAATTTTTGCAAATTATCA      4561
RP      CGGTTAGTACTCTATCACTAGACATAATAATACCCAATTCTTAATTTTTGCAAATTATCA      4561
TK      CGGTTAGTACTCTATCACTAGACATAATAATACCCAATTCTTAATTTTTGCAAATTATCA      4561
FF      CGGTTAGTACTCTATCACTAGACACAATAATACCCGATTCTTAATTTTTGCAAATTATCA      4547
SD      CGGTTAGTACTCTATCACTAGACACAATAATACCCGATTCTTAATTTTTGCAAATTATCA      4620
        ************************ ********** ************************

FP      ATATTTCGATTAGTTACTTGTATTCCTATTAAAAAACATATTTTTGATAAGAAATCATTT      4621
RP      ATATTTCGATTAGTTACTTGTATTCCTATTAAAAAACATATTTTTGATAAGAAATCATTT      4621
TK      ATATTTCGATTAGTTACTTGTATTCCTATTAAAAAACATATTTTTGATAAGAAATCATTT      4621
FF      ATATTTCGATTAGTTACTTGTATTCCTATTAAAAAACATATTTTTTATAAGAAATTATTT      4607
SD      ATATTTCGATTAATTACTTGTATTCCTATTAAAAAACATTTTTTTGATAAGAAATCATTT      4680
        ************ ************************** ***** ********* ****

FP      AATCTTAAATAAAATCAATTCTAAGAAAAAACAAGTCGTCTCTAAGGTGTCCAGTGACGT      4681
RP      AATCTTAAATAAAATCAATTCTAAGAAAAAACAAGTCGTCTCTAAGGTGTCCAGTGACGT      4681
TK      AATCTTAAATAAAATCAATTCTAAGAAAAAACAAGTCGTCTCTAAGGTGTCCAGTGACGT      4681
FF      AATCTTAAATAAAATCAATTCTAAGAAAAAATAAGTCGTCTCTAAGGTGTCCAGTGACGT      4667
SD      AATCTTAAATAAAATCAATTATAAGA-AAAATAAGTCGTCTTTAAGGTGTCCAGTGACGT      4739
        ******************** ***** **** ********* ******************

FP      AGTACAGCGTACCTACAAATTTCTCATCAATTTTGTAATGGAAATCTATGTGATCATAAC      4741
RP      AGTACAGCGTACCTACAAATTTCTCATCAATTTTGTAATGGAAATCTATGTGATCATAAC      4741
TK      AGTACAGCGTACCTACAAATTTCTCATCAATTTTGTAATGGAAATCTATGTGATCATAAC      4741
FF      ACTACAGCGTACCTACAAATTTCTCATCAACTTTGTAATGGAAATCTATGTGATCATAAC      4727
SD      AGTACCGCGTACCTACAAATTTCTCATCAATTTTGTAATGGAAATCTATGTGATCATAAC      4799
        * *** ************************ *****************************
```

```
FP      TATGTACCCAAATAAAGTTAGAGAATAAATACATACTTT---------------------      4780
RP      TATGTACCCAAATAAAGTTAGAGAATAAATACATACTTT---------------------      4780
TK      TATGTACCCAAATAAAGTTAGAGAATAAATACATACTTT---------------------      4780
FF      TATGTACCCAAATAAAGTTAGAGAATAAATACATACTTTCAGGGGCGAACCCATAAATAA      4787
SD      TATGTACCCAAATAAAGTTAGAGAATAAATACATACTTT---------------------      4838
        ***************************************

FP      ------------------------------------------------------------      4780
RP      ------------------------------------------------------------      4780
TK      ------------------------------------------------------------      4780
FF      AATTTTGGGGTAGCGAAAATTTTTTTGATGTTGTTTTACACTTTTTAAAAAAATTGGGGT      4847
SD      ------------------------------------------------------------      4838

FP      ------------------------------------------------------------      4780
RP      ------------------------------------------------------------      4780
TK      ------------------------------------------------------------      4780
FF      GGCAAAAGTCGATAATCTTAATCATTTTTTGGGAATTTGGGGTAGCGAGTGCTACCCTTT      4907
SD      ------------------------------------------------------------      4838

FP      --------------------------------GACCATTCAGTCATAGCTAAATTTT---      4805
RP      --------------------------------GACCATTCAGTCATAGCTAAATTTT---      4805
TK      --------------------------------GACCATTCAGTCATAGCTAAATTTT---      4805
FF      AGCTACTATGTAGGTTCCCCCATGCATACTTTGACCATTCAGTCATAGCTAAATTTT---      4964
SD      --------------------------------GACCATTCAGTCATAGCTAAATTTTATG      4866
                                        *************************

FP      ------------GTCAGTTCTATACTTAGAAAATTTACTTAACATAGTAAGTAAAAAAGC      4853
RP      ------------GTCAGTTCTATACTTAGAAAATTTACTTAACATAGTAAGTAAAAAAGC      4853
TK      ------------GTCAGTTCTATACTTAGAAAATTTACTTAACATAGTAAGTAAAAAAGC      4853
FF      ------------GTCAGTTCTATACTTAGAAAATTTACTTAACATAGTAAGTAAAAAAGC      5012
SD      TTAAGGTGTATAGTCAGTTCTATACTTGGATAATTTACTTAACATAGTAAGTAAAAAAGC      4926
                    *************** ** *****************************

FP      AAATGTTCATATGACAAGAAAAAAA-TTTGACTTAATGGTA-TAAAAATTACCATTTTAA      4911
RP      AAATGTTCATATGACAAGAAAAAAA-TTTGACTTAATGGTA-TAAAAATTACCATTTTAA      4911
TK      AAATGTTCATATGACAAGAAAAAAA-TTTGACTTAATGGTA-TAAAAATTACCATTTTAA      4911
FF      AAGTGTTCATATGACAAGAAAAAAAA-TTGACTAAATGGTAA---------CCATTTTAA      5062
SD      AAA---------------AAAAAAAA-TTGACTAAATGGTAATAAAAATTACCATTTTAA      4970
        **                *******  ****** *******          *********

FP      ATAAAGTTCTTGAAATTTAATAAATAA-AA--CAAGGAAAAAGATAAGGGCTGTATTTAA      4968
RP      ATAAAGTTCTTGAAATTTAATAAATAA-AA--CAAGGAAAAAGATAAGGGCTGTATTTAA      4968
TK      ATAAAGTTCTTGAAATTTAATAAATAA-AA--CAAGGAAAAAGATAAGGGCTGTATTTAA      4968
FF      ATAAAATTCTTGAAATTTAATAAATAAGAATT----------------------------      5094
SD      ATAAAATTCTTGAAATTTAATAA-------------------------------------      4993
        ***** *****************

FP      AGTGTCAAAAAAGGAATGAAAATCTTAAATCAAGCATTAATGACATCAATTTACGCGTAA      5028
RP      AGTGTCAAAAAAGGAATGAAAATCTTAAATCAAGCATTAATGACATCAATTTACGCGTAA      5028
TK      AGTGTCAAAAAAGGAATGAAAATCTTAAATCAAGCATTAATGACATCAATTTACGCGTAA      5028
FF      ------------------------------------------------------------      5094
SD      ------------------------------------------------------------      4993

FP      TTGTGTCTTAAATTTCTAATTTAATTCCATTTTGGGAAGTAATTTAGTTCTTAAATACAG      5088
RP      TTGTGTCTTAAATTTCTAATTTAATTCCATTTTGGGAAGTAATTTAGTTCTTAAATACAG      5088
TK      TTGTGTCTTAAATTTCTAATTTAATTCCATTTTGGGAAGTAATTTAGTTCTTAAATACAG      5088
FF      ------------------------------------------------------------      5094
SD      ------------------------------------------------------------      4993
```

```
FP      CCCTAAGGATTGATTTATCATTACCGATAAAACAAAAG-TTGTAATTTAGTAATTTATTT      5147
RP      CCCTAAGGATTGATTTATC-TTACCGATAAAACAAAAG-TTGTAATTTAGTAATTTATTT      5146
TK      CCCTAAGGATTGATTTATCATTACCGATAAAACAAAAGTTTGTAATTTAGTAATTTATTT      5148
FF      --------------------------ATAAAACAAAAG-TTGTAATTTAGTAATTTATTT      5127
SD      --------------------------ATAAAACAAAAG-TTGTAATTTAGTAATTTATTT      5026
                                  ************ *********************

FP      ACATTGGATTCATAGATTTTGTATGATGATTAAGACTTAAGTACTTAACTAATTAAGATG      5207
RP      ACATTGGATTCATAGATTTTGTATGATGATTAAGACTTAAGTACTTAACTAATTAAGATG      5206
TK      ACATTGGATTCATAGATTTTGTATGATGATTAAGACTTAAGTACTTAACTAATTAAGATG      5208
FF      ACATTGGATTCATAGATTTTGTACGATGATTAAGACTTAAGTACTTAACTAATTAAGATG      5187
SD      ACATTGGATTCATAGATTTTGTATGATGATTAAGACTTAAGTACTTAACTAATTAAGATG      5086
        *********************** ************************************

FP      TAGGGTTTAGTTGGTAGTTAGATGTATGTGTCAGATTTAATTAATTTGACACCTA---GC      5264
RP      TAGGGTTTAGTTGGTAGTTAGATGTATGTGTCAGATTTAATTAATTTGACACCTA---GC      5263
TK      TAGGGTTTAGTTGGTAGTTAGATGTATGTGTCAGATTTAATTAATTTGACACCTA---GC      5265
FF      TAGAGTTTAGTTGGTAGTTAGATGTATGTGTCAGATTTAATTAATTTAACACCTAGTAGC      5247
SD      TAGGGTATAGTTGGTAGTTAGATGTATATGTCAGATTTAATTAATTTGACACCTA---GC      5143
        *** ** ******************** ******************* *******   **

FP      TTTTATTAAATAAATACTATATATATAAGCCTGTTCTTCCTTTTGGTATTTTTGGTATTG      5324
RP      TTTTATTAAATAAATACTATATATATAAGCCTGTTCTTCCTTTTGGTATTTTTGGTATTG      5323
TK      TTTTATTAAATAAATACTATATATATAAGCCTGTTCTTCCTTTTGGTATTTTTGGTATTG      5325
FF      TTATATTAAATAAATACTATATATATAAGCTTGTTCTTCCTTTTTGTATTTTTGGTATTG      5307
SD      TTATAATAAATAAATACTATATATATAAGCTTGTTCTTCCTTTTGGTATTTTTGGTATTG      5203
        ** ** ************************ ************* ***************

FP      TGATGTCATGAATTTTGAATTATTATGTTATATGGCAATTCATCTTTCTTTATTTGGAAG      5384
RP      TGATGTCATGAATTTTGAATTATTATGTTATATGGCAATTCATCTTTCTTTATTTGGAAG      5383
TK      TGATGTCATGAATTTTGAATTATTATGTTATATGGCAATTCATCTTTCTTTATTTGGAAG      5385
FF      TGATGTCATGAATTTTGAATTATTATGTTATATGACAATTCATCTTTCTTTATTTGGAAA      5367
SD      TGATGTCATGAATTTTTAATTATTATGTTATATGACAATTCATCTTTCTTTATTTGGAAA      5263
        **************** ***************** ************************

FP      TTAGTATTTAAACTAGGTTCGACCCCGTGCATTAAATGAATGTCTTATAGAGTTATTTTT      5444
RP      TTAGTATTTAAACTAGGTTCGACCCCGTGCATTAAATGAATGTCTTATAGAGTTATTTTT      5443
TK      TTAGTATTTAAACTAGGTTCGACCCCGTGCATTAAATGAATGTCTTATAGAGTTATTTTT      5445
FF      TTAGTATTTAA-------------------------------------------------      5378
SD      --AGAATTTAA-------------------------------------------------      5272
          *********

FP      ATTTCTACTAAATTATATATATAATACTGGTATCTCATTAGTTGTTCATTTTTCTCGTCA      5504
RP      ATTTCTACTAAATTATATATATAATACTGGTATCTCATTAGTTGTTCATTTTTCTCGTCA      5503
TK      ATTTCTACTAAATTATATATATAATACTGGTATCTCATTAGTTGTTCATTTTTCTCGTCA      5505
FF      ------------------------------------------------------------      5378
SD      ------------------------------------------------------------      5272

FP      ATACATTTTGTTTTGATAATTAAAAAGTTGAAACTGAAAATTAATTAAGAGAATTGAGTA      5564
RP      ATACATTTTGTTTTGATAATTAAAAAGTTGAAACTGAAAATTAATTAAGAGAATTGAGTA      5563
TK      ATACATTTTGTTTTGATAATTAAAAAGTTGAAACTGAAAATTAATTAAGAGAATTGAGTA      5565
FF      ------------------------------------------ATTTGCTATGATATGTTA      5396
SD      ------------------------------------------------------------      5272

FP      GCATGTTGAAATGTATTTTTGAAAAAGAATATTTTTTTATATCGCAAAACTAATGATTCC      5624
RP      GCATGTTGAAATGTATTTTTGAAAAAGAATATTTTTTTATATCGCAAAACTAATGATTCC      5623
TK      GCATGTTGAAATGTATTTTTGAAAAAGAATATTTTTTTATATCGCAAAACTAATGATTCC      5625
FF      GCAAGTCTCTCATAAAACCGTCTTAGTCAGTCACTTTACATTCGTTTCACGCTTACAAGT      5456
SD      ------------------------------------------------------------      5272
```

```
FP      AATTTAATTTTTCTCAATTTTTTGCCTCGACAGTAATTAAAATGGTTTATAATTTTGTAT     5684
RP      AATTTAATTTTTCTCAATTTTTTGCCTCGACAGTAATTAAAATGGTTTATAATTTTGTAT     5683
TK      AATTTAATTTTTCTCAATTTTTTGCCTCGACAGTAATTAAAATGGTTTATAATTTTGTAT     5685
FF      TACAACCTTAGTAAATAAGAATTTTTCACATTGTTTGCAAAATTTCTGTATGTAGTCGGT     5516
SD      ------------------------------------------------------------     5272

FP      TATAGCTAGTATGTGTTAAATTATTCTCAAATAATAGCATCAGTCAAATATTTAGTAATT     5744
RP      TATAGCTAGTATGTGTTAAATTATTCTCAAATAATAGCATCAGTCAAATATTTAGTAATT     5743
TK      TATAGCTAGTATGTGTTAAATTATTCTCAAATAATAGCATCAGTCAAATATTTAGTAATT     5745
FF      GTTTGCAATGGCAGACCGGTAGACAATAACATTTTGAAACGATTTTAAGATTTGAATTTG     5576
SD      ------------------------------------------------------------     5272

FP      CATAGTTTGATACAATTTTTTTTATATTTAAATTAAATGATTATAGATTATAGTTAGTCA     5804
RP      CATAGTTTGATACAATTTTTTTTATATTTAAATTAAATGATTATAGATTATAGTTAGTCA     5803
TK      CATAGTTTGATACAATTTTTTTTATATTTAAATTAAATGATTATAGATTATAGTTAGTCA     5805
FF      GAGTTTATTTAGAGTACAAGATGTATTGTGGTTTTAATTAGTCGCAATTCTAAGATATTT     5636
SD      ------------------------------------------------------------     5272

FP      AAAAAATATAATTTGATGAAAAGATTAATTTTAATGAAAAGATAATAATTTAATAAAATA     5864
RP      AAAAAATATAATTTGATGAAAAGATTAATTTTAATGAAAAGATAATAATTTAATAAAATA     5863
TK      AAAAAATATAATTTGATGAAAAGATTAATTTTAATGAAAAGATAATAATTTAATAAAATA     5865
FF      TTTCAAAGTCGAGAGGTGGACGACACAATTAACTTTATGTTAATAAGGTCATGGGTCGAA     5696
SD      ------------------------------------------------------------     5272

FP      AATTATTATAATTATTAGCTTCCTTACTTAGTTAATGTATACATAATTGATATTAGCTAC     5924
RP      AATTATTATAATTATTAGCTTCCTTACTTAGTTAATGTATACATAATTGATATTAGCTAC     5923
TK      AATTATTATAATTATTAGCTTCCTTACTTAGTTAATGTATACATAATTGATATTAGCTAC     5925
FF      ATAAGGAGATAATGAGATACAAAATGAGACGCTGAACAAAAATTCGGATTTTAGGCGGCT     5756
SD      ------------------------------------------------------------     5272

FP      TTTATTTAGAAAGATGTTTTTTAGAGGAAAAATTTGTGAAGATGCCTATTAGTAAATAGA     5984
RP      TTTATTTAGAAAGATGTTTTTTAGAGGAAAAATTTGTGAAGATGCCTATTAGTAAATAGA     5983
TK      TTTATTTAGAAAGATGTTTTTTAGAGGAAAAATTTGTGAAGATGCCTATTAGTAAATAGA     5985
FF      AGGTGCACATTATGCACTAGTACTCCATAATGTTTACCTTTGACATATTACATAGGTTCG     5816
SD      ------------------------------------------------------------     5272

FP      GGATTTGCTATGATATGTTAGCAAGTCTCTCTTAAAACCGTCTTAGTAATAAGTCGATTT     6044
RP      GGATTTGCTATGATATGTTAGCAAGTCTCTCTTAAAACCGTCTTAGTAATAAGTCGATTT     6043
TK      GGATTTGCTATGATATGTTAGCAAGTCTCTCTTAAAACCGTCTTAGTAATAAGTCGATTT     6045
FF      GAAAATTTTGATGTTTTTTAGCAAGTCTCTCTTAAAACCGTCTTAGTAATAAGTCGATTT     5876
SD      --ATTTGCTATGATATGTTAGCAAGTCTCTCTTAAAATCGTCTTAGTCA---GTCACTTT     5327
          *  *  *    * * ******************** ********* *   ***  ***

FP      ACATTCGTTTCACCCTG-CAAGTTACAACCGTAGTAAATAAGAATTTCGAAGGATATGTC     6103
RP      ACATTCGTTTCACCCTG-CAAGTTACAACCGTAGTAAATAAGAATTTCGAAGGATATGTC     6102
TK      ACATTCGTTTCACCCTG-CAAGTTACAACCGTAGTAAATAAGAATTTCGAAGGATATGTC     6104
FF      ACATTCGTTTCACCCTG-CAAGTTACAACCGTAGTAAATAAGAATTTCGAAGGATATGTC     5935
SD      ACATTCGTTTCACTCTTACAAGTTACAACCTGTCAACCTTAGTAAATAAGAAT-------     5380
        ************* **  ************     *  * ** *  *   *

FP      ATA--------TGTGTCATATATGAGCTCTTGTGTGGATGACCTATGAGCTTATTTTGCA     6155
RP      ATA--------TGTGTCATATATGAGCTCTTGTGTGGATGACCTATGAGCTTATTTTGCA     6154
TK      ATA--------TGTGTCATATATGAGCTCTTGTGTGGATGACCTATGAGCTTATTTTGCA     6156
FF      ATATACTCATATGTGTCATACATGAGCTCTTGTGTGGATGACCTATGAGCTTATTTTGCA     5995
SD      -----------------------------------------------------TTTT-CA     5386
                                                             **** **
```

```
FP      CATTGTTTACAAAATTTC-GTATGTAGTCGGTGTTTGCAATGCTAGACCGGTAGACAATA      6214
RP      CATTGTTTACAAAATTTC-GTATGTAGTCGGTGTTTGCAATGCTAGACCGGTAGACAATA      6213
TK      CATTGTTTACAAAATTTC-GTATGTAGTCGGTGTTTGCAATGCTAGACCGGTAGACAATA      6215
FF      CATTGTTTACAAAATTTC-GTATGTAGTCGGTGTTTGCAATGGTAGACCGGTAGACAATA      6054
SD      CATTGTTTGCAAAATTTCTGTATGTAGTCGGTGTTTGCAATGGCAGACCGGTAGACAATA      5446
        ******** ********* ***********************  ****************

FP      ACATTTTGAAACGATTTTAAGATTTGAATTTGGAGTTTATTTAGAGTACAAGATGTATTG      6274
RP      ACATTTTGAAACGATTTTAAGATTTGAATTTGGAGTTTATTTAGAGTACAAGATGTATTG      6273
TK      ACATTTTGAAACGATTTTAAGATTTGAATTTGGAGTTTATTTAGAGTACAAGATGTATTG      6275
FF      AAGTTTTGAAACGATTTTAAGATTTGAATTTGGAGTTTATTTAGAGTTCAAGATGTATTG      6114
SD      ACATTTTGAAACGATTTTAAGATTTGAATTTGGAGTTTATTTAGAGTACAAGATGTATTG      5506
        *  ******************************************** ************

FP      TGGTTTTAATTAGTCGCAATTTTAAGATATTTTTTCAAAGTCGAGAGATGGACGAC----      6330
RP      TGGTTTTAATTAGTCGCAATTCTAAGATATTTTTTCAAAGTCGAGAGATGGACGAC----      6329
TK      TGGTTTTAATTAGTCGCAATTCTAAGATATTTTTTCAAAGTCGAGAGATGGACGAC----      6331
FF      TGGTTTTAATTAGTCGCAATTCTAAGATATTTTTTCAAAGTCGAGAGGTGGACGACACTC      6174
SD      TGGTTTTAATTAGTCGCAATTTTAAGATATTTTTTCAAAGTCGAGAGGTGGACGAC----      5562
        ******************** ************************** ********

FP      ---ACAATTAACTTTATGTTAATAGGGTCATGGGTCGAAATAAGGAGATAATGAGATCCA      6387
RP      ---ACAATTAACTTTATGTTAATAGGGTCATGGGTCGAAATAAGGAGATAATGAGATACA      6386
TK      ---ACAATTAACTTTATGTTAATAGGGTCATGGGTCGAAATAAGGAGATAATGAGATACA      6388
FF      GACACAATTAACTTTTTGTTAATAGGGTCATGGGTCGAAATAAGGAGATAATGAGATACA      6234
SD      ---ACAATTAACTTTATGTTAATTGGGTC-TGGGTCGAAATAAGGAGATAATGAGATACA      5618
           ************ ******* ***** *************************** **

FP      AAATGAGACGCAGAACAAAAATTCGGATTTTAGGCGGCTAGGTGCACATTATGCACAAGT      6447
RP      AAATGAGACGCAGAACAAAAATTCGGATTTTAGGCGGCTAGGTGCACATTATGCACAAGT      6446
TK      AAATGAGACGCAGAACAAAAATTCGGATTTTAGGCGGCTAGGTGCACATTATGCACAAGT      6448
FF      AAATGAGACGCAGAATGAAAATTCGG--------------GGTGCACATTATGCACTAGT      6280
SD      AAATGAGACGCAGAACAAAAATTCGGATTTTAGGCGGCTAGGTGCACATTATGCACTAGT      5678
        ***************  *********              **************** ***

FP      ACTCCATAATGTTTACCTTTGACATATTACCTAGGTTCGGAAAATTTTGATTTGCTTTGG      6507
RP      ACTCCATAATGTTTACCTTTGACATATTACCTAGGTTCGGAAAATTTTGATTTGCTTTGG      6506
TK      ACTCCATAATGTTTACCTTTGACATATTACCTAGGTTCGGAAAATTTTGATTTGCTTTGG      6508
FF      ACTCCATAATGTTTACCTTTGACATATTACATAGGTTCGGAAAAAATT---TTGATTTGC      6337
SD      ACTCCATAATGTTTACCTTTTACATATTACCTAGATTCGGACAATTTTGATTTGCTTTGG      5738
        ******************** ********* *** ****** **  **   *** ****

FP      AGAAGCGAAAACAAACGCTTCACTCGGTCCCCATTCCC----------------------      6545
RP      AGAAGCGAAAACAAACGCTTCACTCGGTCCCCATTCCC----------------------      6544
TK      AGAAGCGAAAACAAACGCTTCACTCGGTCCCCATTCCC----------------------      6546
FF      AGAAAGGAAAACAAACGCTTCACTCGATCCCCATTCCCCGACCTACTAAT----------      6387
SD      AGAAAGGAAAACAAACGCTTCACTCGGTCCCCTTTCCCTGACCTGCTAATCAAAAGAGCA      5798
        ****  ******************** ***** *****

FP      ------------------------------------------------------------      6545
RP      ------------------------------------------------------------      6544
TK      ------------------------------------------------------------      6546
FF      ------------------------------------------------------------      6387
SD      TTTTTTTGTGATGACGAGGGGGTTGAATCCCCCGGGCCCATGCATTCCCGCACCACCACA      5858

FP      ------------------------------------------------------------      6545
RP      ------------------------------------------------------------      6544
TK      ------------------------------------------------------------      6546
FF      ----------------------------------------------CAAAAGAGCAATAT      6401
SD      TGGACTATGTAAGCCACCCCTTTGGGGGCTGCAGTGGCCAAGTGATCAAAAGAGCAATAT      5918
```

```
FP      -----CGACCTTCTTGTTTTGGTTTCGATTTTATGTTTGGAGTGACTAATTAACATTGAT      6600
RP      -----C-ACCTTCTTGTTTTGGTTTCGATTTTATGTTTGGAGTGACTAATTAACATTGAT      6598
TK      -----CGACCTTCTTGTTTTGGTTTCGATTTTATGTTTGGAGTGACTAATTAACATTGAT      6601
FF      TGTCCTTGCCTTCTTGTTTTGGTTTCGATTTTATGTTTAGAGTGACTAATTAACATTGAT      6461
SD      TGTTCTTACCTTCTTGTTTTGGTTTCGATTTTATGTTAGGAGTGACTAATTAACAGTGAT      5978
                *****************************  **************** ****

FP      TAGCTGCAGCGTCAAGGGTACCATAGACCGGTACAAGAAAGCCAGCTCCGACAACTCTGG      6660
RP      TAGCTGCAGCGTCAAGGGTACCATAGACCGGTACAAGAAAGCCAGCTCCGACAACTCTGG      6658
TK      TAGCTGCAGCGTCAAGGGTACCATAGACCGGTACAAGAAAGCCAGCTCCGACAACTCTGG      6661
FF      TAGCTGCAGCGTCAAGGGTACCATAGACCGGTACAAGAAAGCCAGCTCCGACAACTCTGG      6521
SD      TAGCTGCAGCGTCAAGGGTACCATAGACCGGTACAAGAAAGCCAGCTCCGACAACTCTGG      6038
        ************************************************************

SL               CGTCAAGGGTACCATAGACCGGTACAAGAAAGCCTCCTCCGACAACTCTGG

FP      TGCCAGCTCTGCTGCTGAAGCCAATGCTCAGGTACCCTTTTCCACCCTCTTCAAGACTAG      6720
RP      TGCCAGCTCTGCTGCTGAAGCCAATGCTCAGGTACCCTTTTCCACCCTCTTCAAGACTAG      6718
TK      TGCCAGCTCTGCTGCTGAAGCCAATGCTCAGGTACCCTTTTCCACCCTCTTCAAGACTAG      6721
FF      TGCCAGCTCTGCTGCTGAAGCCAATGCTCAGGTACCCTTTTCCACCCTCTTCAAGACTAG      6581
SD      TGCCAGCTCTGCTGCTGAAGCCAATGCTCAGGTACCCTTTTCCACCCTCTTCAAGACTAG      6098
        ************************************************************

SL      TGCCAGCTCTGCTGCTGAAGCCAATGCTCAG

FP      GGTAAAGGCCGCGAACATCTAAGCCCCTAGCTCGTCATTTGTGACAATCTTTAAGACATT      6780
RP      GGTAAAGGCCGCGAACATCTAAGCCCCTAGCTCGTCATTTGTGACAATCTTTAAGACATT      6778
TK      GGTAAAGGCCGCGAACATCTAAGCCCCTAGCTCGTCATTTGTGACAATCTTTAAGACATT      6781
FF      GGTAAAGGCCGCGAACATCTAAGCCCCTAGCTCGTCATTTGTGACAATCTTTAAGACATT      6641
SD      GGTAAAGGCCGCGAACATCTAAGCCCTTAGCTCGTCATTTGTGACAATCTTTAAGACATT      6158
        ************************** *********************************

FP      AAGACGATAATGTTGTTGTTTATGTTCGATAAATGTTGCAGTACTATCAACAAGAAGCTG      6840
RP      AAGACGATAATGTTGTTGTTTATGTTCGATAAATGTTGCAGTACTATCAACAAGAAGCTG      6838
TK      AAGACGATAATGTTGTTGTTTATGTTCGATAAATGTTGCAGTACTATCAACAAGAAGCTG      6841
FF      AAGACGATAATGTTGTTGTTTATGTTCGATAAATGTTGCAGTACTATCAACAAGAAGCTG      6701
SD      AAGACGATAATGTTGTTGTTTATGTTCGATAAATGTTGCAGTACTATCAACAAGAAGCTG      6218
        ************************************************************

SL                                               TACTATCAACAAGAAGCTG

FP      CTAAATTGAGGAACCAGATCCGTACAGTAACAGAGAATAACAGGTAAATTTCATAGGATA      6900
RP      CTAAATTGAGGAACCAGATCCGTACAGTAACAGAGAATAACAGGTAAATTTCATAGGATA      6898
TK      CTAAATTGAGGAACCAGATCCGTACAGTAACAGAGAATAACAGGTAAATTTCATAGGATA      6901
FF      CTAAATTGAGGAACCAGATCCGTACAGTAACAGAGAATAACAGGTAAATTTCATAGGATA      6761
SD      CTAAATTGAGGAACCAGATCCGTACAGTAACAGAGAATAACAGGTAAATTTCATAGGATA      6278
        ************************************************************

SL      CTAAATTGAGGAACCAGATCCGTACAGTAACAGAGAATAACAGGTAAATTTCATAGGATA

FP      AAAATGTAAGCAATTTGACGCAGTAAATATTAACATAGTTACTGAGACACAAAAAAAGGT      6960
RP      AAAATGTAAGCAATTTGACGCAGTAAATATTAACATAGTTACTGAGACACAAAAAAAGGT      6958
TK      AAAATGTAAGCAATTTGACGCAGTAAATATTAACATAGTTACTGAGACACAAAAAAAGGT      6961
FF      AAAATGTAAGCAATTCGACGAAGTAAATATTGACATAGTTACTGAGACACAAAAAAAGGT      6821
SD      AAAATGTAAGCAATTCGACGAAGTAAATATTAACATAGTTACTGAGACACAAAAAAAGGT      6338
        *************** **** ********** ****************************

SL      AAAATGTAAGCAATTCGACGCAGTAAATATTAACATAGTTACTGAGACACAAAAAAAGGT

FP      TGCTTTCCAGGCATTTGATGGGTGAAGGCCTAAGCAGTCTAAAGTCTAAACATGAAGGAT      7020
RP      TGCTTTCCAGGCATTTGATGGGTGAAGGCCTAAGCAGTCTAAAGTCTAAACATGAAGGAT      7018
TK      TGCTTTCCAGGCATTTGATGGGTGAAGGCCTAAGCAGTCTAAAGTCTAAACATGAAGGAT      7021
FF      TGCTTTCCAGGCATTTGATGGGTGAAGGCCTAAGCAGTCTA-------AACATGAAGGAT      6874
SD      TGCTTTCCAGGCATTTGATGGGTGAAGGCCTAAGCAGTCTA-------AACATGAAGGAT      6391
        *****************************************       ************

SL      TGCTTTCCAGGCATTTGATGGGTGAAGGCCTAAGCAGTCTA-------AACATGAAGGAT
```

```
FP      CTCAAGAGCCTTGAGAATAAACTTGAAAGAGGAATTAGCCGAATCCGATCTAAAAAGGTA      7080
RP      CTCAAGAGCCTTGAGAATAAACTTGAAAGAGGAATTAGCCGAATCCGATCTAAAAAGGTA      7078
TK      CTCAAGAGCCTTGAGAATAAACTTGAAAGAGGAATTAGCCGAATCCGATCTAAAAAGGTA      7081
FF      CTTAAGAGTCTTGAGAATAAACTTGAAAGAGGAATTAGCCGAATCCGATCTAAAAAGGTA      6934
SD      CTTAAGAGCCTTGAGAATAAACTTGAAAGAGGAATTAGCCGAATCCGATCTAAAAAGGTA      6451
        ** ***** ***************************************************

SL      CTTAAGAGCCTTGAGAATAAACTTGAAAGAGGAATTAGCCGAATCCGATCTAAAAAG

FP      CTCCCT--------------------------TATATCTCAGTAATCTCCGACTTGATTA      7114
RP      CTCCCT--------------------------TATATCTCAGTAATCTCCGACTTGATTA      7112
TK      CTCCCT--------------------------TATATCTCAGTAATCTCCGACTTGATTA      7115
FF      CTCCCTTTACTTGATTAAACCTTAATCGCTCTTACAGTTCAGTAATCTCCGACTTGATTA      6994
SD      CTCCCTTTACTTGATTAAACCTTAATCGCTCTTACAGTTCAGTAATCTCCGACTTGATTA      6511
        ******                          ** *  **********************

FP      TGAACACGATTATTATGAAGTTGACAATGTTTTAATTTGTGTCTTATAGAATGAGCTGC      7174
RP      TGAACACGATTATTATGAAGTTGACAATGTTTTAATTTGTGTCTTATAGAATGAGCTGC      7172
TK      TGAACACGATTATTATGAAGTTGACAATGTTTTAATTTGTGTCTTATAGAATGAGCTGC      7175
FF      TGAACACGATAATTATGAAGTTGATGATGTTTTAATTTGTGTCTGATAGAATGAGCTGC      7054
SD      TGAACACGATAATTATGAAGTTGATGAGGTTTTAATTTGTGTCTTATAGAATGAGCTGC      6571
        ********** *************  * **************** ***************

SL                                                        AATGAGCTGC

FP      TGTTTGCTGAGATTGAGTTCATGCAGAAAAGGGTAAGTCTTCTGTTTCCTTAATCGCATT      7234
RP      TGTTTGCTGAGATTGAGTTCATGCAGAAAAGGGTAAGTCTTCTGTTTCCTTAATCGCATT      7232
TK      TGTTTGCTGAGATTGAGTTCATGCAGAAAAGGGTAAGTCTTCTGTTTCTTTAATCGCATT      7235
FF      TGTTTGCTGAGATTGAGTTCATGCAGAAAAGGGTAACTCTTCTGTTTCCTTAATCGCATT      7114
SD      TGTTTGCTGAGATTGAGTTCATGCAGAAAAGGGTAAGTCTCCTGTTTCCTTAATCGCATT      6631
        ************************************ *** ******* ***********

SL      TGTTTGCTGAGATCGAGTTCATGCAGAAAAGG

FP      CAATCTTAAGTTGAAATCGTCATGCCTCTTTACATTATAGTTACTCTTGTGTAAGACG-G      7293
RP      CAATCTTAAGTTGAAATCGTCATGCCTCTTTACATTATAGTTACTCTTGTGTAAGACG-G      7291
TK      CAATCTTAAGTTGAAATCGTCATGCCTCTTTACATTATAGTTACTCTTGTGTAAGACG-G      7294
FF      CAATCTTAAGTTGAAATCGTCATGCCTCCATACATTACAGTTGGTCTTGCATAAGACGGG      7174
SD      CAATCTTAAGTTGAAATCGTCATGCCTCCATACATTACAGTTGGTCTTGCATAAGACGGG      6691
        ****************************  ******* ****  *****  ******* *

FP      TTTTGTCTGATGCTTCTTATAAGTTAAGAAGTACTTGTTATTGCTATCATTGACTGAAAT      7353
RP      TTTTGTCTGATGCTTCTTATAAGTTAAGAAGTACTTGTTATTGCTATCATTGACTGAAAT      7351
TK      TTTTGTCTGATGCTTCTTATAAGTTAAGAAGTACTTGTTATTGCTATCATTGACTGAAAT      7354
FF      TTTTATCTGATGCTT----TAAGTTATGGAGTAGTTATTATTGCTATCATTGACTGAAGT      7230
SD      TTTTATCTGATGCTTCTTATAAGTTATGGAGTACTTATTATTGCTATCATTGACTGAAGT      6751
        **** **********    ******* * **** ** ********************* *

FP      GTATAACGCATAACACGGGATAAGGCCATTGTACTCTATACAACGGTCTTATTTGCGACC      7413
RP      GTATAACGCATAACACGGGATAAGGCCATTGTACTCTATACAACGGTCTTATTTGCGACC      7411
TK      GTATAACGCATAACACGGGATAAGGCCATTGTACTCTATACAACGGTCTTATTTGCGACC      7414
FF      GTATAACGAATAACACCGGATAAGGCCATTGTACTCTATACAACGGTCTTATTTGCGACT      7290
SD      GTATAACGAATAACACGGGATAAGGCCATTGTACTCTATACAACGGTCTTATTTGCGACT      6811
        ******** ******* ****************************************** 

FP      AGACCTGACAAAATCAACCCGACATGACCCGAAAATGCTGACCTGAGATCCGAATTCAAC      7473
RP      AGACCTGACAAAATCAACCCGACATGACCCGAAAATGCTGACCTGAGATCCGAATTCAAC      7471
TK      AGACCTGACAAAATCAACCCGACATGACCCGAAAATGCTGACCTGAGATCCGAATTCAAC      7474
FF      AGACCTAGCAAAATCAACCCGAC------------------CC-GAGATCCGAAATCGAC      7331
SD      AGACCTAGCAAAATCAACCCGAT-----CCGAATTT---------------GAAATCGAC      6851
        ******  **************                             *** ** **

FP      CCGAATTGCCGCACTCGAATGACATCTGAAGCCCGAATTGACCCAACCCGACCCAAAAAT      7533
RP      CCGAATTGCCGCACTCGAATGACATCTGAAGCCCGAATTGACCCAACCCGACCCAAAAAT      7531
TK      CCGAATTGCCGCACTCGAATGACATCTGAAGCCCGAATTGACCCAACCCGACCCAAAAAT      7534
FF      CCGAACTGCCGCACCCGAATGACATCCAAAGCCTGAATTGACCCAACTC-----AAAAAT      7386
SD      CCGAACTGCCGCACCCGAATGACATCCGAAGCCCGAATTGACCCAACTCAACCCAAAAAT      6911
        ***** ******** ***********  ***** ************* *     ******
```

```
FP      GACCCGA----------------------------------------------CCCCAAA    7547
RP      GACCCGA----------------------------------------------CCCCAAA    7545
TK      GACCCGA----------------------------------------------CCCCAAA    7548
FF      GGCCCGAGCTTTCATAGACCCGACCCGAAATGACCCGATTTGAAACCACCCAACCCGAAA    7446
SD      GACCCGAGCTTTCATAGACCCGACCCGATCTA----------AAACCACCCA--------    6953
        * *****

FP      TGACCCGATCTGAAACCACCCAACCCGAAAATGACCCGACAAAACATAACTTTAATTGAC    7607
RP      TGACCCGATCTGAAACCACCCAACCCGAAAATGACCCGACAAAACATAACTTTAATTGAC    7605
TK      TGACCCGATCTGAAACCACCCAACCCGAAAATGACCCGACAAAACATAACTTTAATTGAC    7608
FF      TGACTCGATTTGAAACCACCCAACCCGAAAATGACCCGACAAAACATAACTTTAATTGAT    7506
SD      --ACCCGATCTAAAACCACCCAACCCGAAAATGACCAGACAAACCATAACTTTAATTGAC    7011
          ** **** * ************************ ****** ***************

FP      CCCAAAAGACTTGAAATGCGTTTTTTTTTTCTTCTCTTAATCATTG-ACACGAAAATAAC    7666
RP      CCCAAAAGACTTGAAATGCGTTTTTTTTTTCTTCTCTTAATCATTG-ACACGAAAATAAC    7664
TK      CCCAAAAGACTTGAAATGCGTTTTTTTTTTCTTCTCTTAATCATTG-ACACGAAAATAAC    7667
FF      CCCAAAAGACTTGAAATGCGTTTTTTT------CTCTTAATCATTGGACCCGAAAATGCC    7560
SD      CCCAAAAGACTTGAAATGCGTTTTTTTT-----CTCTTAATCATTG-ACCCGAAAATGAT    7065
        ***************************      ************* ** *******

FP      CCGGCTCGAAACAACCCGACCCGCTTGACCCGAAAA-TGACCCAACAAACATACCCGAAA    7725
RP      CCGGCTCGAAACAACCCGACCCGCTTGACCCGAAAA-TGACCCAACAAACATACCCGAAA    7723
TK      CCGGCTCGAAACAACCCGACCCGCTTGACCCGAAAA-TGACCCAACAAACATACCCGAAA    7726
FF      CAGGTCCGAAACAACCTGACCCGCTTGACCCGAAAA-TGACCCAACAAACATACCCGAAA    7619
SD      CCGGACCGAAACAACCCGACCCGCTTGACCCAAAAAATGACCCAACAAACATACCCAAAA    7125
        * **  ********** ************** **** ******************* ***

FP      GGACCCGACCCGA-CCGAACTAACCCGATAATGTGATAAACCTGAGCAGACCTGACCTGA    7784
RP      GGACCCGACCCGA-CCGAACTAACCCGATAATGTGATAAACCTGAGCAGACCTGACCTGA    7782
TK      GGACCCGACCCGA-CCGAACTAACCCGATAATGTGATAAACCTGAGCAGACCTGACCTGA    7785
FF      GGACCCGACCCGAACCGAACTAACCCGATAATGTGGCAAACC-GAGTAGACCTGACTCGA    7678
SD      GGACCCGACCCGA-CCG-ACTAACCCGATATTTTGGTAAACCCGAGCAGACCCGACCCGA    7183
        ************* *** ************ * **  ***** *** ***** ***  **

FP      CCCGACCTGACCTGACCCGAGCCCGACCCGTTGATCCATTTTCCCAGGTCTA--TTTGCG    7842
RP      CCCGACCTGACCTGACCCGAGCCCGACCCGTTGATCCATTTTCCCAGGTCTA--TTTGCG    7840
TK      CCCGACCTGACCTGACCCGAGCCCGACCCGTTGATCCATTTTCCCAGGTCTA--TTTGCG    7843
FF      -----ATTGCCCTGACCCCAACCCGGCCTGTTGACCCATTTTGCCAGGTCTA--TTTGCG    7731
SD      ------GTGGTCTGA-CCGAACCCGGCCCGTTGACCCATTTTGCCAGGTCTAAAATTGCG    7236
               **  **** ** * **** ** ***** ******* *********   *****

FP      ACTATAACATAATGTGGGTTGATCCTGCAGGAAGTAGAACTGCACAACAACAACCAGTTT    7902
RP      ACTATAACATAATGTGGGTTGATCCTGCAGGAAGTAGAACTGCACAACAACAACCAGTTT    7900
TK      ACTATAACATAATGTGGGTTGATCCTGCAGGAAGTAGAACTGCACAACAACAACCAGTTT    7903
FF      ACTATAACATAATGTGGGTTGATCCTGCAGGAAGTAGAACTGCACAACAACAACCAGTTT    7791
SD      ACTATAACATAATGTGGGTTGAT-CTGCAGGAAGTAGAACTGCATAACAACAACCAGTTT    7295
        *********************** ******************** ***************

SL                                    GAAGTAGAACTACACAACAACAACCAGTAT

FP      CTTAGAGCAAAGGTTAGTCTCCTTTTTTTTCT------ATGGTGATCCATCTTCGGTAGT    7956
RP      CTTAGAGCAAAGGTTAGTCTCCTTTTTTTTCT------ATGGTGATCCATCTTCGGTAGT    7954
TK      CTTAGAGCAAAGGTTAGTCTCCTTTTTTTTCT------ATGGTGATCCATCTTCGGTAGT    7957
FF      CTTAGAGCAAAGGTTAGTCTCCTTTTTTTTTTTTTTTTATTGTGATCCATCGTGCGTAGT    7851
SD      CTTAGAGCAAAGGTTAGTCTCCTTTTTTATTTTTTTTTCTTGTGATCCATTTTGCGTAGT    7355
        ****************************  * *      * *********  *  *****

SL      CTTAGAGCAAAG

FP      TTGCTGCTATTT---GTTGCGACATACGGAGTAATTAGTTGATATAAACAAAATGATGGT    8013
RP      TTGCTGCTATTT---GTTGCGACATACGGAGTAATTAGTTGATATAAACAAAATGATGGT    8011
TK      TTGCTGCTATTT---GTTGCGACATACGGAGTAATTAGTTGATATAAACAAAATGATGGT    8014
FF      TTGCTGCTATTTTTCGTTGCGACATACGGAGTAATTAATTGATATAAACAAAATGATGGT    7911
SD      TTGCTGCTATTT---GTTGCGACATACGGAGTAATTAGTTGATATAAACAAAATGATGGT    7412
        ************   ********************** **********************
```

```
FP      TGAATTACATAGCAGATAGCAGAAAATGAGAGAGCACAACA-------------------      8054
RP      TGAATTACATAGCAGATAGCAGAAAATGAGAGAGCACAACA-------------------      8052
TK      TGAATTACATAGCAGATAGCAGAAAATGAGAGAGCACAACA-------------------      8055
FF      TGAATTACATAGCAGATAGCAGAAAATGAGAGAGCACAACAGGTTGAATTACATAGCAGA      7971
SD      TGAATTACATAGCAGATAGCAGAAAATGAGAGAGCACAACA-------------------      7453
        *****************************************
SL                     ATAGCAGAAAATGAGAGAGCACAACA-------------------

FP      -------------------------AAGTATGAGCTTGATGCCAGGTGGAAGCAACGAGT      8089
RP      -------------------------AAGTATGAGCTTGATGCCAGGTGGAAGCAACGAGT      8087
TK      -------------------------AAGTATGAGCTTGATGCCAGGTGGAAGCAACGAGT      8090
FF      TAGCAGAAAATGAGAGAGCACAACAAAGTATGAGCTTGATGCCAGGTGGAAGCAGCGAGT      8031
SD      -------------------------AAGTATGAGCTTGATGCCAGGTGGAAGCAGTGAGT      7488
                                 *****************************  ****
SL      -------------------------AAGTATGAGCTTGATGCCAGGTGGAAGCAGTGAGT

FP      ATGAGCTGGCACCGCCACCCCAGTCATTCGACTCCCGGAATTACTTCCAAGTCAATGCAC      8149
RP      ATGAGCTGGCACCGCCACCCCAGTCATTCGACTCCCGGAATTACTTCCAAGTCAATGCAC      8147
TK      ATGAGCTGGCACCGCCACCCCAGTCATTCGACTCCCGGAATTACTTCCAAGTCAATGCAC      8150
FF      ATGAGCTGGCACCGCCACCTCAGTCATTCGACTCCCGGAATTACTTCCAAGTCAATGCTC      8091
SD      ATGAGCTGGCACCGCCACCTCAGTCATTCGACTCCCGGAATTACTTCCAAGTCAATGCTC      7548
        ******************* ************************************** *
SL      ATGAGCTGGCACCGCCACCTCAGTCATTCGACTCCCGGAATTACTTCCAAGTCAATGCTC

FP      TTCAACCCAATAATGCCCACTACTCTCGCCCAGACCAGACAACTCTCCAGCTTAACTAA      8208
RP      TTCAACCCAATAATGCCCACTACTCTCGCCCAGACCAGACAACTCTCCAGCTTAACTAA      8206
TK      TTCAACCCAATAATGCCCACTACTCTCGCCCAGACCAGACAACTCTCCAGCTTAACTAA      8209
FF      TTCAACCCAATAATAACCACTACTCTCGCCCAGACCAGACAACTCTCCAGCTTAACTAA      8150
SD      TTCAACCCAATAATAACCACTACTCTCGCCCAGACCAGACAACTCTCCAGCTTAACTAA      7607
        **************  *******************************************
SL      TTCAACCCAATAATAACCACTACTCTCGCCCAGACCAGACAACTCTCCAGCTTAACTAA
```
